# Dysregulation of RAS proteostasis by autosomal-dominant *LZTR1* mutation induces Noonan syndrome–like phenotypes in mice

**DOI:** 10.1172/jci.insight.182382

**Published:** 2024-11-22

**Authors:** Taiki Abe, Kaho Morisaki, Tetsuya Niihori, Miho Terao, Shuji Takada, Yoko Aoki

**Affiliations:** 1 Department of Medical Genetics, Tohoku University School of Medicine, Sendai, Japan.; 2 Department of Systems BioMedicine, National Research Institute for Child Health and Development, Tokyo, Japan.

**Keywords:** Development, Genetics, Drug therapy, Genetic diseases, Mouse models

## Abstract

*Leucine-zipper–like posttranslational regulator 1* (*LZTR1*) is a member of the BTB-Kelch superfamily, which regulates the RAS proteostasis. Autosomal dominant (AD) mutations in *LZTR1* have been identified in patients with Noonan syndrome (NS), a congenital anomaly syndrome. However, it remains unclear whether *LZTR1* AD mutations regulate the proteostasis of the RAS subfamily molecules or cause NS-like phenotypes in vivo. To elucidate the pathogenesis of *LZTR1* mutations, we generated 2 *LZTR1* mutation knock-in mice (*Lztr1*^G245R/+^ and *Lztr1*^R409C/+^), which correspond to the human p.G248R and p.R412C mutations, respectively. LZTR1-mutant male mice exhibit low birth weight, distinctive facial features, and cardiac hypertrophy. Cardiomyocyte size and the expression of RAS subfamily members, including MRAS and RIT1, were significantly increased in the left ventricles (LVs) of mutant male mice. LZTR1 AD mutants did not interact with RIT1 and functioned as dominant-negative forms of WT LZTR1. Multi-omics analysis revealed that the mitogen-activated protein kinase (MAPK) signaling pathway was activated in the LVs of mutant mice. Treatment with the MEK inhibitor trametinib ameliorated cardiac hypertrophy in mutant male mice. These results suggest that the MEK/ERK pathway is a therapeutic target for the NS-like phenotype resulting from dysfunction of RAS proteostasis by *LZTR1* AD mutations.

## Abstract 2

Autosomal dominant mutations in LZTR1 causes dysregulation of RAS proteostasis and induces Noonan syndrome-like phenotypes in a novel mouse model.

## Graphical abstract

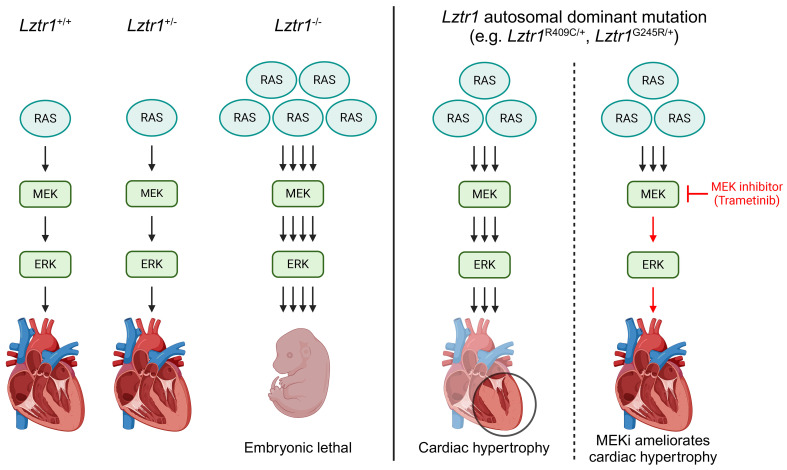

## Introduction

Noonan syndrome (NS; OMIM #163950 and #605275) is a malformation syndrome and a type of RASopathy. RASopathies are a family of syndromes caused by germline mutations in genes encoding various components of the RAS/mitogen-activated protein kinase (MAPK) signaling pathway (1, 2). RASopathies include NS, Costello syndrome, and cardio-facio-cutaneous (CFC) syndrome. NS is characterized by congenital heart disease, low birth weight, short stature, distinctive facial appearance, skeletal abnormalities, intellectual disability, and other specific phenotypes (3). *Protein tyrosine phosphatase 11* (*PTPN11*), which encodes SHP2, *SOS Ras/Rac guanine nucleotide exchange factor 1* (*SOS1*), *Raf-1 proto-oncogene, serine/threonine kinase* (*RAF1*), and *Ras-like without CAAX 1* (*RIT1*) are the major pathogenic genes of NS.

**Figure 1:**
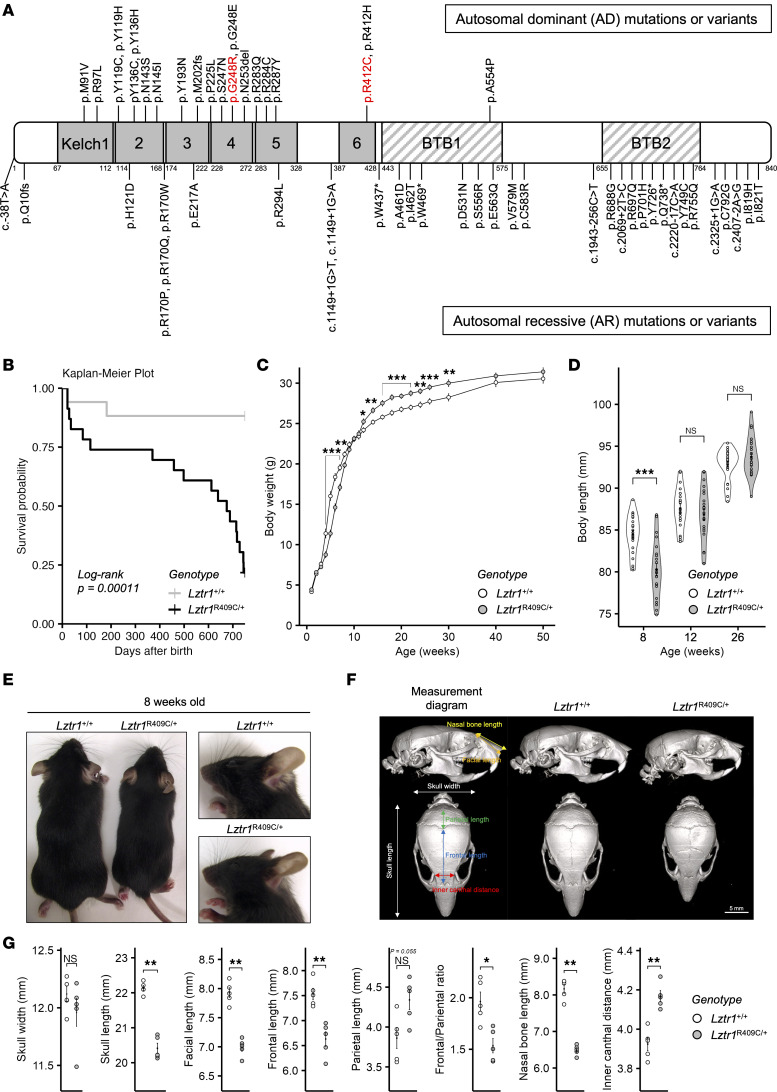
*Lztr1*^R409C/+^ mice show phenotypes similar to those in patients with NS. (**A**) A schematic structure of the representative *LZTR1* mutations and variants. The numbers under the bar denote the amino acid positions. Variants indicated above the bar are autosomal dominant mutations, whereas variants below the bar are autosomal recessive mutations. The mutations in red (p.G248R and p.R412C) correspond to the mutated sites in the mouse models generated in this study. (**B**) Kaplan-Meier survival curves of male *Lztr1*^R409C/+^ (*n* = 23) and *Lztr1*^+/+^ (*n* = 17) mice were generated by using R packagesggplot2 and survminer. (**C**) Chronological changes in body weight were calculated at the indicated time points for 1 year. (**D**) Chronological changes in body length measured at 8, 12, and 26 weeks of age. (**E**) Representative images of *Lztr1*^R409C/+^ and *Lztr1*^+/+^ male mice at 8 weeks of age. (**F** and **G**) Representative reconstructed μCT images of skulls from *Lztr1*^R409C/+^ and *Lztr1*^+/+^ male mice at 8 weeks of age (*n* = 5). Scale bar: 5 mm. Image reconstruction and various measurements were performed using 3D Slicer imaging software. Values are presented as the mean ± SEM. *P ≤ 0.05, **P ≤ 0.01, ***P ≤ 0.001, using Wilcoxon–Mann–Whitney test.

*Leucine-zipper–like posttranslational regulator 1* (*LZTR1*) is a tumor suppressor gene and one of the known causative genes of NS (4). Germline and somatic mutations in *LZTR1* have been identified in patients with NS, schwannomatosis, and various cancers, including glioblastoma, one of the most challenging cancers to treat (5–7). LZTR1 belongs to the BTB-Kelch superfamily and functions as an adaptor protein of the Cullin 3–based (CUL3-based) ubiquitin E3 ligase complex. Members of the BTB-Kelch superfamily homo- or heterodimerize and interact with CUL3 via the BTB domain. The Kelch domain traps substrates for mono- or polyubiquitination. LZTR1 binds to RAS subfamily members, including RIT1, MRAS, and KRAS. Trapped RAS proteins are ubiquitinated and proteolyzed by the proteasome or have altered cellular localization (8–11). Almost all gene mutations responsible for NS are autosomal dominant (AD). However, pathogenic mutations in *LZTR1* have 2 hereditary forms: AD and autosomal recessive (AR). Patients with *LZTR1* AR mutations exhibit a more severe phenotype than those with *LZTR1* AD mutations in NS (12, 13). Most *LZTR1* AD mutations are located in the Kelch domain, whereas biallelic AR mutations have been identified in the BTB domains, Kelch domain, and intronic regions that regulate transcriptional activity or increase minor transcriptional variants (Figure 1A) (4, 12, 14–25). According to previous in vitro mutational analyses, pathogenic *LZTR1* mutations in patients with NS and schwannomatosis have been considered loss-of-function mutations, regardless of whether they are AD or AR hereditary forms. Intriguingly, homozygous *Lztr1*-deficient mice (*Lztr1*^–/–^) exhibit embryonic lethality, whereas heterozygous *Lztr1*-deficient mice (*Lztr1*^+/–^) exhibit mild or no specific phenotypes, including cardiac hypertrophy and facial appearance (10, 26). A recent study demonstrated that LZTR1 p.L580P, an AR variant located in the BTB domain, polymerizes and alters its subcellular localization (27). This study provides insights into the functional differences between *LZTR1* deletions and AR variants. Based on previous reports, we hypothesized that *LZTR1* AD mutants lose the ability to interact with RAS subfamily members and function as dominant-negative forms of LZTR1 WT, resulting in high expression of RAS subfamily members and activation of the MAPK signaling pathway.

LZTR1 was first identified as a transcription-related molecule (28). Subsequent studies have revealed that it directly regulates the ubiquitination of the RAS subfamily members (8–11, 17), epidermal growth factor receptor (EGFR) (29), AXL receptor (29), and charged multivesicular protein 1B (30). Additionally, LZTR1 indirectly regulates the ubiquitination of SEC31A by interacting with Kelch-like protein 12 (KLHL12), a member of the BTB-Kelch superfamily (31). These functional interactions have been explored using LZTR1-KO cell lines and LZTR1-deficient mice but have not been validated in pathological mutation models. Moreover, no in vivo evidence has demonstrated whether AD mutations in *LZTR1* cause RAS accumulation, leading to NS-like phenotypes, including congenital heart defects.

To address these questions, we generated 2 potentially novel genetically modified mice with heterozygous *LZTR1* AD mutations. Here, we show that *LZTR1* AD mutations caused NS-like phenotypes and that LZTR1 AD mutants functioned as dominant-negative forms of WT. Furthermore, treatment with the MEK inhibitor trametinib ameliorated congenital heart defects in a potentially novel NS mouse model. Our findings provide insights into the pathogenicity of RASopathies and the development of therapeutic drugs for these diseases.

## Results

### LZTR1 AD mutant mice show NS-like phenotypes.

To reveal the pathogenic mechanism of NS in patients with heterozygous *LZTR1* AD mutations, we generated 2 types of *Lztr1*–knock-in mice harboring the p.R409C and p.G245R mutations, which are inherited human AD mutations corresponding to p.R412C and p.G248R, respectively (Figure 1A and Supplemental Figure 1; supplemental material available online with this article; https://doi.org/10.1172/jci.insight.182382DS1) (4, 14, 15, 17, 21, 32). First, we examined the survival rate and chronological changes in body weight and length during a long-term follow-up study. Approximately 25% of *Lztr1*^R409C/+^ mice survived for 2 years. The mice began to die suddenly after 1 year of age without any obvious signs, including loss of body weight, food consumption, or physical activity (Figure 1B). The body weights and lengths of *Lztr1*^R409C/+^ mice were lower than those of the *Lztr1*^+/+^ mice until approximately 10 weeks of age, after which they caught up with the *Lztr1*^+/+^ mice (Figure 1, C and D). Analyzing the facial appearance and skeletal abnormalities via μCT, *Lztr1*^R409C/+^ mice showed smaller and rounder skulls, more blunt snouts, and hypertelorism compared with their *Lztr1*^+/+^ littermates (Figure 1, E–G). Furthermore, *Lztr1*^R409C/+^ mice exhibited splenomegaly and renal hypertrophy (Supplemental Figure 2A), consistent with those found in other RASopathy mouse models, including HRAS p.G12S and RIT1 p.A57G (33, 34). H&E staining demonstrated extramedullary hematopoiesis in the spleens and livers of *Lztr1*^R409C/+^ mice (Supplemental Figure 2B). Splenomegaly may be caused by extramedullary hematopoiesis.

*Lztr1*^R409C/+^ pups were not born at the expected Mendelian ratio, suggesting that the heterozygous AD mutation in *Lztr1* causes partial embryonic lethality (Supplemental Figure 3A). Embryonic analysis revealed that *Lztr1*^R409C/+^ embryos had edema and/or s.c. hemorrhage compared with their *Lztr1*^+/+^ littermates on E14.5 and E16.5 (Supplemental Figure 3C). The homozygous mutation in *Lztr1* (*Lztr1*^R409C/R409C^) was embryonically lethal (Supplemental Figure 3B). *Lztr1*^R409C/R409C^ embryos exhibited more severe edema and s.c. hemorrhage than *Lztr1*^R409C/+^ mice (Supplemental Figure 3C). Subsequently, we performed whole-mount immunostaining of the embryonic back skin to assess lymphatic phenotypes (Supplemental Figure 3D). The immunostaining using an antibody against the lymphatic endothelial cell surface marker VEGFR3 showed that *Lztr1*^R409C/+^ mutant embryos had lymphatic abnormalities compared with WT littermates. Moreover, *Lztr1*^R409C/R409C^ mutant embryos exhibited more severe phenotypes in terms of lymphatic morphology. These results suggest that lymphatic defects might be one of the reasons for embryonic lethality in homozygous mutant embryos. Similar phenotypes were observed in *Lztr1*^G245R/+^ and *Lztr1*^G245R/G245R^ embryos (Supplemental Figure 3, E and F). Taken together, these results are similar to those reported in other mouse models of RASopathy (33, 34).

### LZTR1 AD mutants function as dominant-negative forms to LZTR1 WT.

Our results suggest that heterozygous *LZTR1* AD mutations cause NS-like phenotypes. However, the differences between AD mutations and the gene deletions that mimic compound heterozygous AR mutations remain unclear. Previous studies have reported different results regarding the phenotypes of *Lztr1*-deficient mice. A group reported that *Lztr1*^–/–^ C57BL/6 mice showed embryonic lethality, whereas *Lztr1*^+/–^ C57BL/6 mice are born yet show cardiac hypertrophy (10). In contrast, another group demonstrated that *Lztr1*^+/–^ C57BL/6 mice did not display any specific phenotypes related to heart frailty; however, *Lztr1*^–/–^ 129S1/SvImJ mice (129 mice) are viable yet show cardiac hypertrophy and facial disability similar to those in patients with NS (26). In our experiments, *Lztr1*^+/–^ C57BL/6 mice did not show cardiac hypertrophy or a high expression of RAS subfamily members (Supplemental Figure 4, A and B). A high expression of the RAS subfamily members was detected in *Lztr1*^–/–^ mouse embryonic fibroblasts (MEFs) but not in *Lztr1*^+/–^ MEFs (Supplemental Figure 4C). Our results and those of previous studies indicate that a haploinsufficiency of *LZTR1* did not cause NS.

**Figure 2:**
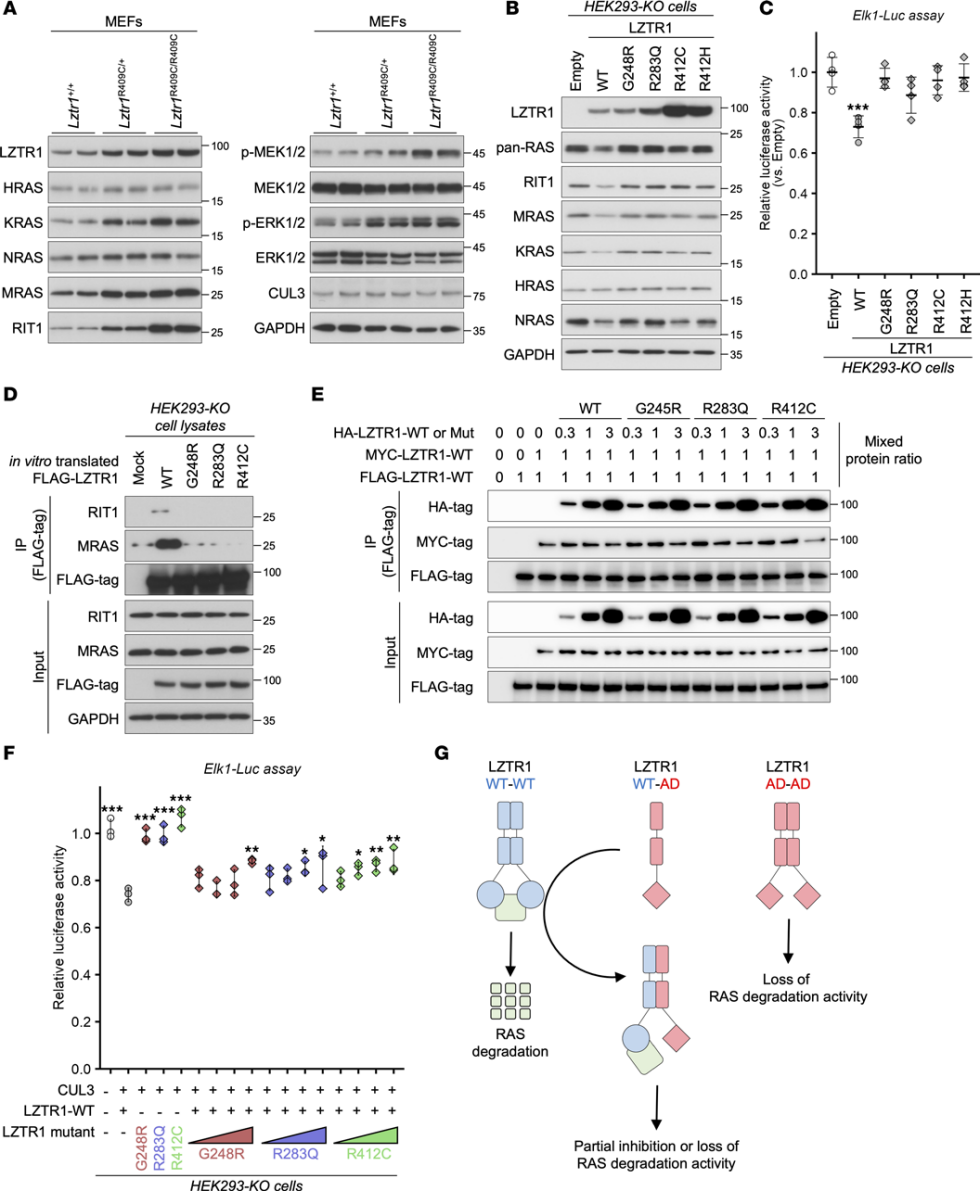
Autosomal dominant LZTR1 mutants act as dominant-negative forms of the WT LZTR1. (**A**) Mouse embryonic fibroblasts (MEFs) from *Lztr1*^+/+^, *Lztr1*^R409C/+^, and *Lztr1*^R409C/R409C^ embryos at E13.5 were analyzed using the indicated antibodies. (**B**) HEK293-KO cells were transfected with plasmids encoding WT or autosomal dominant (AD) mutants. After 48 hours, whole-cell lysates were evaluated via immunoblot analysis using each anti-RAS subfamily antibody. (**C**) HEK293-KO cells were transfected with the pFR-luc transreporter, pFA2-ELK1, pGL4.74-hRluc-TK, and the indicated expression plasmids. The Elk1-mediated transcriptional activities were evaluated under 10% serum conditions and displayed as relative values, with the empty group as a control. Values are presented as mean ± SD (*n* = 4). ***P ≤ 0.001 (versus the empty group, using Dunnett’s test). (**D**) In vitro–translated FLAG-LZTR1 WT or the indicated mutants and HEK293-KO cell lysates were incubated with FLAG-M2 magnetic beads for 12 hours, and immunoprecipitants were evaluated by immunoblotting. (**E**) In vitro translated FLAG-, MYC-, and HA-tagged LZTR1 WT or AD mutant proteins were incubated with FLAG-M2 magnetic beads for 6 hours, and the influence of HA-tagged LZTR1 proteins on the homodimerization between FLAG-LZTR1 WT and MYC-LZTR1 WT was evaluated by immunoblotting. (**F**) HEK-293KO cells were transfected with the indicated plasmids, and then the Elk1-mediated transcriptional activities were analyzed as shown in **C**. Values are presented as mean ± SD (*n* = 3). *P ≤ 0.05, **P ≤ 0.01, ***P ≤ 0.001 vs. the CUL3/LZTR1-WT group, using Dunnett’s test. (**G**) Schematic of LZTR1 dimerization and its effect on RAS degradation.

To investigate the molecular events accompanying the *LZTR1* mutation, we generated MEFs and analyzed their expression levels of RAS subfamily members. The expression of RAS subfamily members, particularly KRAS, MRAS, and RIT1, as well as phospho-MEK1/2 and phospho-ERK1/2, was higher in *Lztr1*^R409C/+^ and *Lztr1*^R409C/R409C^ MEFs than in *Lztr1*^+/+^ MEFs (Figure 2A). LZTR1 expression in MEFs increased in the following order: *Lztr1*^+/+^, *Lztr1*^R409C/+^, and *Lztr1*^R409C/R409C^. These results correlated with the expression levels of RAS subfamily members (Figure 2A). In contrast, the LZTR1 p.G245R mutation did not affect LZTR1 protein levels, whereas *Lztr1*^G245R/+^ and *Lztr1*^G245R/G245R^ MEFs exhibited high expression of the RAS subfamily members, similar to MEFs harboring the LZTR1 p.R409C mutant (Supplemental Figure 5). These results suggest that heterozygous AD mutations in *LZTR1* induce the accumulation of RAS subfamily members.

Given the above data, we hypothesized that LZTR1 AD mutants, including human p.R412C/mouse p.R409C and human p.G248R/mouse p.G245R, are not only loss-of-function mutants but also play a dominant-negative role in LZTR1 WT. To verify this hypothesis, we examined the differences and relationships between LZTR1 WT and AD mutants. In LZTR1-KO HEK293 (HEK293-KO) cells, the expression of LZTR1 WT resulted in a decrease in RAS protein levels, which was not observed in cells expressing AD mutants, such as p.G248R and p.R412C (Figure 2B). Under these conditions, the expression levels of the LZTR1 p.R412C mutant were higher than those of the LZTR1 WT or p.G245R mutants; however, the high expression of the LZTR1 p.R412C mutant did not affect RAS protein levels. Subsequently, we performed an ELK1 transactivation assay (*Elk1*-Luc assay) in HEK293-KO cells to assess the downstream signaling activity of the RAS subfamily members. The results show that LZTR1 WT reduced luciferase activity compared with the control group, whereas AD mutants did not alter luciferase activity levels (Figure 2C). An immunoprecipitation assay using in vitro–translated FLAG-LZTR1 proteins demonstrated that LZTR1 WT, not AD mutants, interacted with endogenous MRAS and RIT1 in the absence of endogenous LZTR1 (Figure 2D). In general, members of the BTB-Kelch superfamily function as dimers or multimers to capture their substrates. We observed that HA-LZTR1 AD mutations interacted with FLAG-LZTR1 WT, and homodimerization of FLAG-LZTR1 WT and MYC-LZTR1 WT was interfered with HA-LZTR1 AD mutants in a protein level–dependent manner (Figure 2E). Finally, we tested whether the AD mutants exerted dominant-negative effects on the WT. In the Elk1-Luc assay, AD mutants partially inhibited the WT-dependent downregulation of Elk1 transcriptional activity in a dose-dependent manner (Figure 2F). Taken together, these results suggest that LZTR1 AD mutants lose the ability to interact with RIT1 and function as dominant-negative regulators of the WT protein. It is possible that the AD mutants interfere with the homodimerization of LZTR1 WT and WT, resulting in partial inhibition or complete loss of RAS degradation activity (Figure 2G).

### High expression of the RAS subfamily proteins and activation of the MAPK signaling pathway cause cardiac hypertrophy in LZTR1-mutant mice.

**Figure 3:**
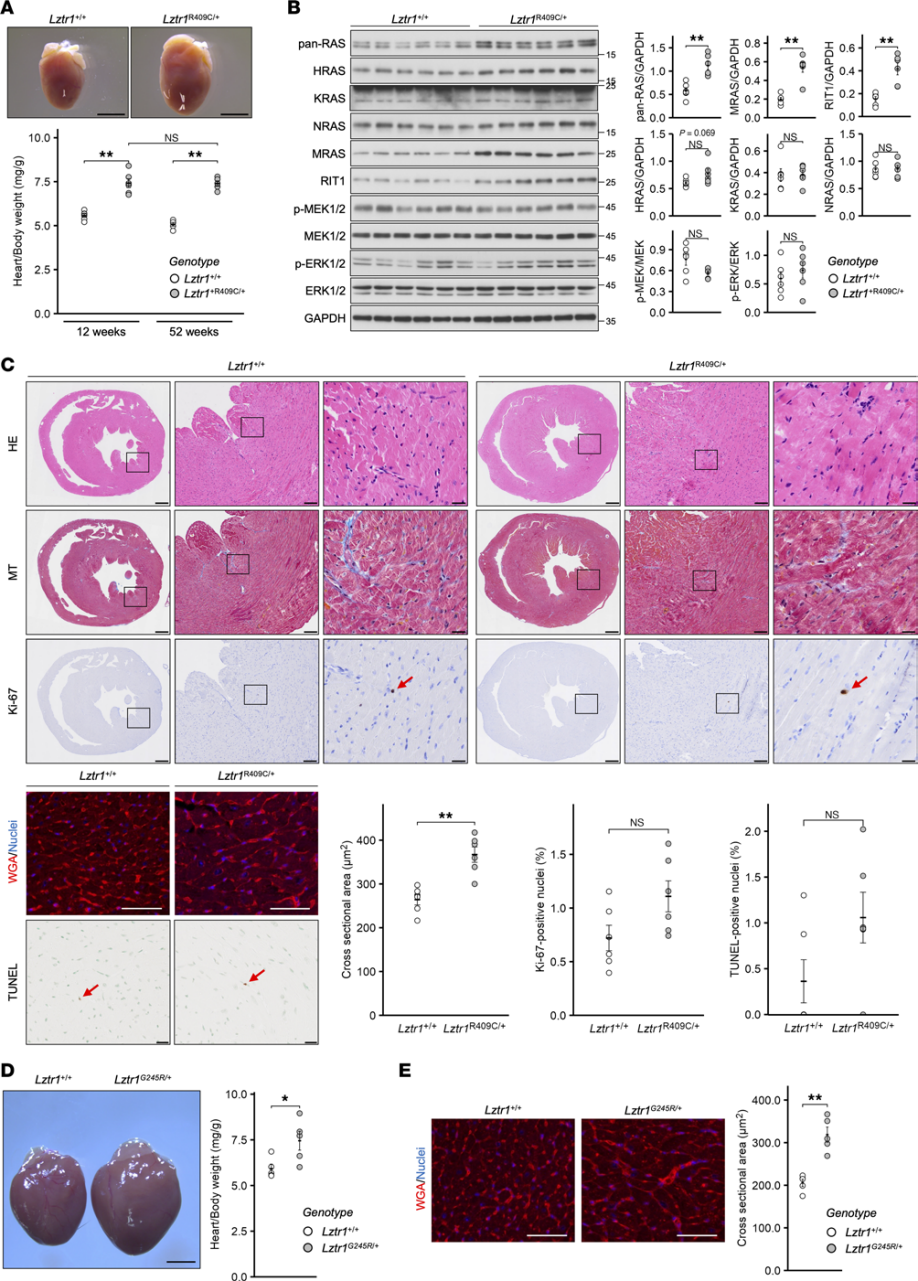
*Lztr1*^R409C/+^ and *Lztr1*^G245R/+^ mice exhibit cardiac hypertrophy and high expression of the RAS subfamily proteins. (**A**) Hearts were collected from *Lztr1*^R409C/+^ and *Lztr1*^+/+^ male mice at 12 and 52 weeks of age. The heart/body weight ratio was calculated. Scale bars: 2 mm. Values are presented as the mean ± SEM (*n* = 6 or 5). (**B**) Left ventricle lysates from 12-week-old mice were analyzed via immunoblotting with the indicated antibodies. Values are presented as the mean ± SEM (*n* = 6). **P ≤ 0.01, using Wilcoxon–Mann–Whitney test. (**C**) Hearts of 12-week-old mice were fixed and embedded in paraffin, and the paraffin-embedded sections were stained with H&E, Masson’s trichrome (MT), anti–Ki-67 antibody (scale bars: 500 μm [left], 100 μm [middle], or 20 μm [right]), WGA (scale bars: 50 μm), and TUNEL staining kit (scale bars: 20 μm). The cross-sectional areas of the WGA^+^ regions and the number of Ki-67^+^ and TUNEL^+^ nuclei were calculated using ImageJ Fiji software. The red arrows show stain-positive nuclei. Values are presented as the mean ± SEM (*n* = 6). **P ≤ 0.01, using Wilcoxon–Mann–Whitney test. (**D** and **E**) Samples were collected from *Lztr1*^G245R+^ and *Lztr1*^+/+^ male mice at 12 weeks of age. The heart/body weight ratio was then calculated. Scale bars: 2 mm (**D**). The cross-sectional areas of the WGA^+^ regions were calculated using ImageJ Fiji software, as in **C**. Scale bars: 50 μm (**E**). Values are presented as the mean ± SEM (*n* = 5). **P ≤ 0.05, **P ≤ 0.01, using Wilcoxon–Mann–Whitney test.

Congenital heart defects have been observed in patients with NS harboring *LZTR1* mutations (15). To investigate the influence of *Lztr1* AD mutation on mouse hearts, we analyzed the hearts of *Lztr1*^R409C/+^ mice at 12 weeks (young adults) and 52 weeks (aged) of age. The heart/body weight ratio of *Lztr1*^R409C/+^ mice was higher than that of their *Lztr1*^+/+^ littermates. These ratios did not change between 12- and 52-week-old mice (Figure 3A), suggesting that the *Lztr1* mutation affects cardiac development at an early stage, from birth to 12 weeks. LZTR1 regulates the ubiquitination and degradation of RAS subfamily proteins. Consequently, the loss of LZTR1 induces RAS accumulation and activates the MAPK signaling pathway in vitro (8, 11). Immunoblot analysis of mouse LVs demonstrated that RAS subfamily members were upregulated in *Lztr1*^R409C/+^ mice, as expected. However, despite the high expression of RAS subfamily members, there was no significant increase in phospho-MEK1/2 and phospho-ERK1/2 levels, as observed in other RASopathy model mice (Figure 3B). The number of Ki-67^+^ nuclei in the left ventricles (LVs) did not increase in 12-week-old *Lztr1*^R409C/+^ mice, whereas wheat germ agglutinin (WGA) staining revealed cardiomyocyte hypertrophy in these mice compared with their *Lztr1*^+/+^ littermates (Figure 3C) (33–35). Furthermore, there were no significant increases in fibrosis or apoptosis in *Lztr1* ^R409C/+^ mouse hearts. Similarly, *Lztr1*^G245R/+^ mice demonstrated cardiomyocyte hypertrophy and a higher expression of RAS subfamily members compared with their *Lztr1*^+/+^ littermates (Figure 3, D and E, and Supplemental Figure 6).

**Figure 4:**
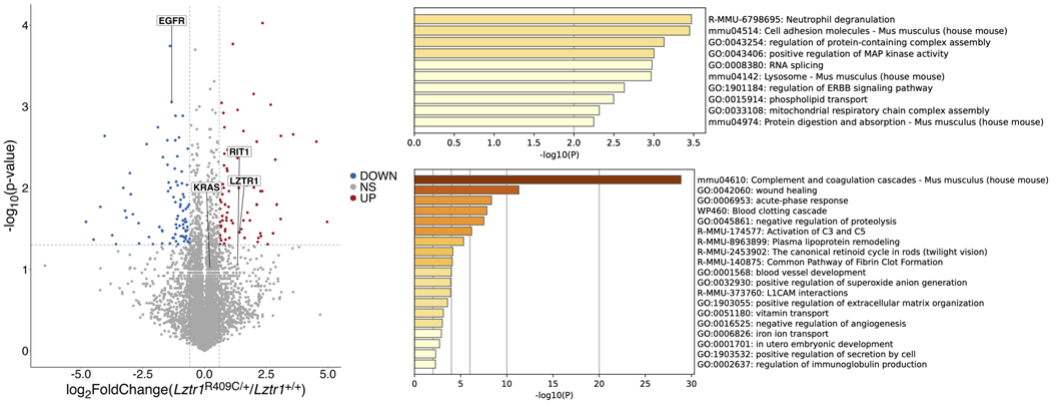
DIA proteome analysis of LVs from *Lztr1*^R409C/+^ and *Lztr1*^+/+^ mice at 12 weeks of age. Volcano plot of DIA proteome analysis data (*n* = 3). For differentially expressed proteins (DEPs), thresholds were defined as an adjusted *P* value less than 0.05 and a log_2_ fold change larger than |0.6|. Upregulated or downregulated proteins are shown in red and blue, respectively. Enrichment analyses of multiple DEPs were performed using Metascape, and the relevant enrichment patterns across multiple protein lists and the top enriched clusters are represented.

To investigate the effect of the LZTR1 mutant on protein instability and gene expression, we performed data-independent acquisition (DIA) proteomic and mRNA-Seq analyses using LVs. We identified 85 downregulated and 72 upregulated proteins in the DIA proteomic analysis (Supplemental Tables 3 and 4). Enrichment analyses of differentially expressed proteins (DEPs) revealed that positive regulation of MAP kinase activity was enriched in the upregulated DEPs group (Figure 4). Upon closer examination of the changes in the expression of LZTR1 target molecules, we observed a 2.5-fold increase in RIT1 (*P* = 0.087) and a 0.4-fold decrease in EGFR (*P* = 0.00087), whereas other RAS members remained unchanged or were undetectable. Interestingly, LZTR1 protein levels were significantly increased in the hearts of *Lztr1*^R409C/+^ mice (2.7-fold, *P* = 0.0346) (Figure 4 and Supplemental Tables 3 and 4). Increased LZTR1 protein expression was also observed in MEFs and transiently transfected HEK293-KO cells (Figure 2, A and B). To compare the protein stability of the LZTR1 mutants, we performed a cycloheximide chase assay (CHX assay). The results of the CHX assay demonstrated that the LZTR1 p.R412C mutant had a longer half-life than the WT and other mutants and that LZTR1 was proteolyzed by the proteasome (Supplemental Figure 7, A and B). The high stability of the LZTR1 p.R412C mutant could be helpful for its dominant-negative effect on LZTR1 WT.

**Figure 5:**
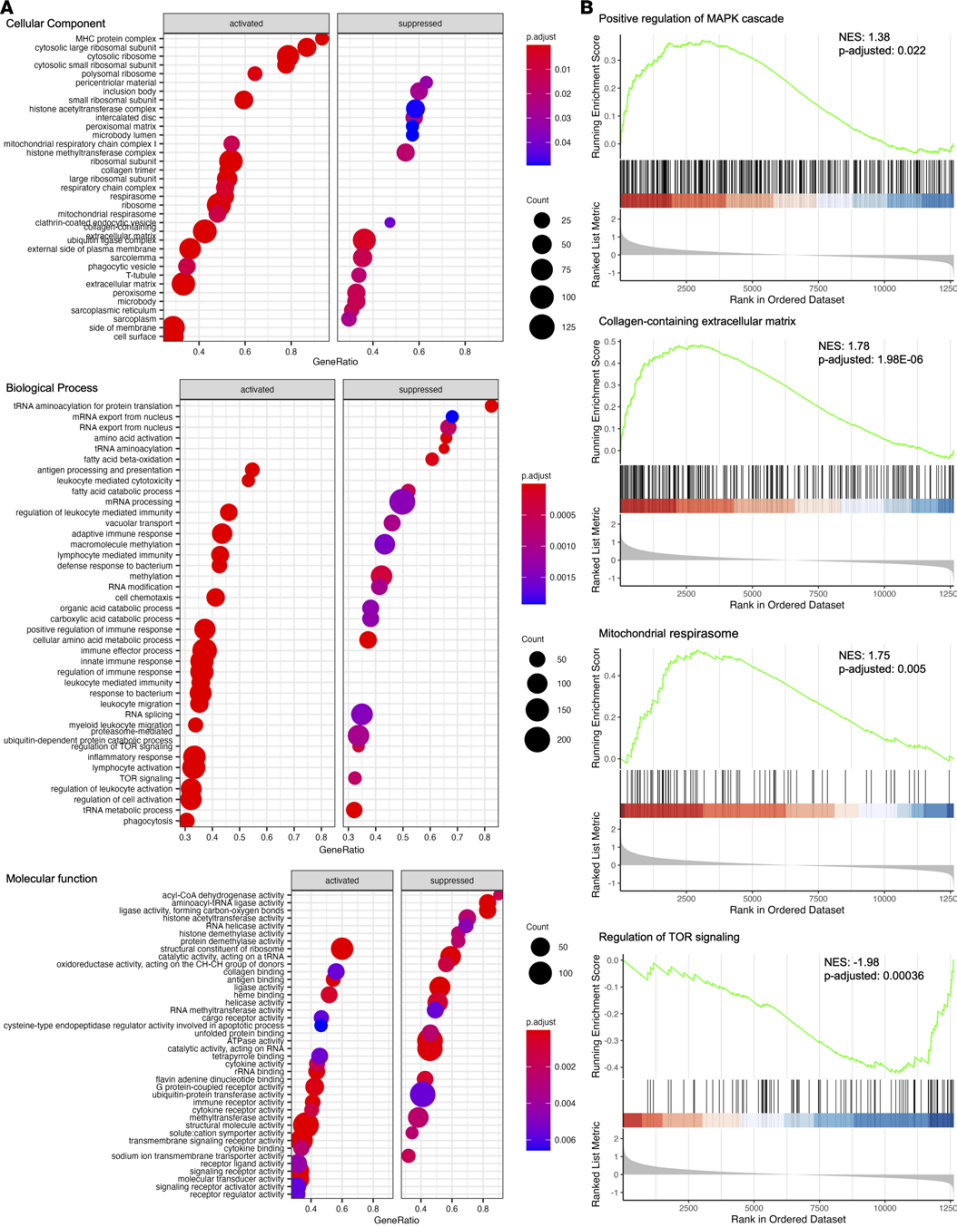
mRNA-Seq demonstrates that autosomal dominant LZTR1 mutant induced the activation of MAPK signaling. mRNA-Seq analyses were performed using RNAs from the left ventricles (*n* = 3), followed by gene ontology (GO) and gene set enrichment analysis (GSEA). (**A**) Representative GO plots, including cellular components, molecular functions, and biological processes. (**B**) Representative GSEA plots showing differentially expressed genes enriched in the indicated gene sets.

The results of mRNA-Seq, gene ontology (GO), and gene set enrichment analyses (GSEA) demonstrate that the MAPK cascade, collagen-containing extracellular matrix, and mitochondrial respirasome-related genes were upregulated in the LVs of *Lztr1*^R409C/+^ mice (Figure 5, A and B, and Supplemental Tables 5 and 6). These GO terms were aligned with known functions of the RAS/MAPK signaling pathway and LZTR1. Conversely, the mTOR signaling pathway, T-tubule, sarcolemma, ubiquitin ligase complex, and fatty acid β-oxidation–related genes were downregulated in LVs from *Lztr1*^R409C/+^ mice (Figure 5, A and B). These results provide evidence that a heterozygous *LZTR1* AD mutation activates the RAS/MAPK signaling pathway in a mouse model.

### Treatment with the MEK inhibitor trametinib ameliorates cardiac hypertrophy in Lztr1^R409C/+^ mice.

**Figure 6:**
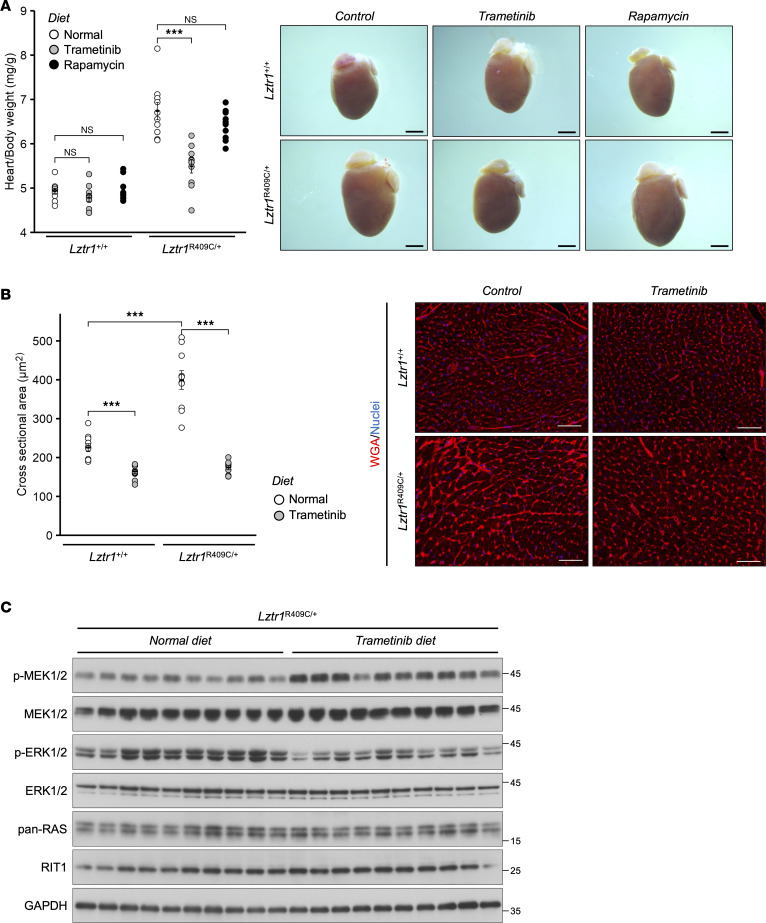
Treatment with the MEK inhibitor trametinib ameliorates cardiac hypertrophy in *Lztr1*^R409C/+^ mice. Mice were fed diets containing 5 ppm trametinib or 10 ppm rapamycin for 8 weeks after weaning, and the collected tissues were used for each analysis. (**A**) The heart/body weight ratio was calculated. Values are presented as the mean ± SEM (*n* = 10). ***P ≤ 0.001, using Wilcoxon–Mann–Whitney test. Scale bars: 2 mm. (**B**) Representative images of wheat germ agglutinin (WGA) staining. The cross-sectional areas of the WGA^+^ regions were calculated using ImageJ software. Scale bars: 50 μm. Values are presented as the mean ± SEM (*n* = 10). ***P ≤ 0.001, using Wilcoxon–Mann–Whitney test. (**C**) Immunoblot analysis of left ventricles from *Lztr1*^R409C/+^ mice was performed with the indicated antibodies.

Symptomatic treatment or surgery is the first-line therapy for patients with NS with congenital heart disease because an effective drug treatment has not yet been established. Longitudinal cohort studies and early-phase clinical trials have identified MEK inhibitors as candidate drugs for RASopathies (36–40). Furthermore, in *Rit1*^A57G/+^ mice, a NS model of cardiac hypertrophy, the AKT/mTOR signaling pathway was activated in tissues from whole embryos and adult hearts under β-adrenergic stimulation (34). Since RIT1 is a principal target of LZTR1 (8, 31), AKT/mTOR signaling inhibitors such as rapamycin may be drug candidates for patients with NS with *LZTR1* mutations. To investigate the therapeutic effects of signal inhibitors, mice were treated with the MEK inhibitor trametinib or a rapamycin-containing diet for 8 weeks after weaning. Compared with the normal diet–fed group, the heart/body weight ratio and cardiomyocyte size decreased in *Lztr1*^R409C/+^ mice fed a trametinib-containing diet (Figure 6, A and B, and Supplemental Figure 8A). Immunoblot analysis revealed that the oral administration of trametinib reduced phospho-ERK levels in the mouse LVs (Figure 6C and Supplemental Figure 8B). However, trametinib treatment did not alleviate splenomegaly or renal hypertrophy in *Lztr1*^R409C/+^ mice (Supplemental Figure 8A). In contrast, rapamycin treatment did not improve specific phenotypes such as cardiac hypertrophy (Figure 6A). Overall, these results suggest that MEK inhibitors will be therapeutic options for patients with NS with AD mutations in *LZTR1*.

## Discussion

In this study, we demonstrate that a heterozygous AD mutation in *LZTR1* caused NS-like phenotypes, including cardiac hypertrophy, low birth weight, short birth stature, and a distinctive facial appearance using AD mutation knock-in mouse models, such as *Lztr1*^R409C/+^ and *Lztr1*^G245R/+^ mice. Our results also show that 3 representative AD mutations lost the ability to interact with RIT1 and function as dominant-negative forms of LZTR1 WT. Furthermore, treatment with the MEK inhibitor trametinib ameliorated cardiac hypertrophy in *Lztr1*^R409C/+^ mice. Our findings provide insights into the pathogenic mechanism of patients with NS with *LZTR1* AD mutations and suggest that MEK inhibitors have the potential to be highly effective drugs against NS.

Research on disease models has shown that signaling inhibitors of the MEK/ERK and AKT/mTOR pathways are therapeutic drug candidates for RASopathies. Clinical trials of these inhibitors are underway in various countries (40–46). A previous study showed that the MEK inhibitor pimasertib, AKT inhibitor ipatasertib, or a combination of these agents could partially rescue embryonic lethality in LZTR1-KO mice (30). However, no evidence suggests that signal inhibitors can ameliorate cardiac hypertrophy in adult mice or humans with LZTR1 pathogenic mutations. In this study, we demonstrated that a heterozygous AD mutation in *LZTR1* activates MAPK signaling and that trametinib treatment ameliorates cardiac hypertrophy in *Lztr1*^R409C/+^ mice (Figure 5 and Figure 6). Conversely, the mTOR signaling pathway was suppressed in *Lztr1*^R409C/+^ mice, and rapamycin treatment did not rescue *LZTR1* mutation–dependent cardiac hypertrophy (Figure 5 and Figure 6). RIT1 and MRAS are the most abundant RAS subfamily members in the LVs of *LZTR1*-mutant mice. MRAS induces RAF activation in a classical RAS–dependent manner, and pathogenic mutations in *MRAS* cause NS with cardiac hypertrophy (47). Recent reports have revealed that the MRAS/SHOC2/PPP1C complex regulates RAF dephosphorylation, resulting in RAF-RAS binding and ERK activation (48–51). Furthermore, MEK inhibition ameliorates cardiomegaly in *Rit1*^M90I/+^ mice (43). Based on these reports and our results, the increase in the expression of RAS subfamily members in the hearts of LZTR1-mutant mice contributes to the activation of MAPK rather than AKT/mTOR signaling. Taken together, although there are issues to be considered in the future, such as the presence or absence of adverse effects associated with long-term drug use, our results provide the first scientific evidence to our knowledge for the role of signaling inhibitors in patients with NS with congenital heart defects carrying heterozygous AD *LZTR1* mutations.

Almost all pathogenic mutations in RASopathies follow AD inheritance patterns, whereas pathogenic mutations in *LZTR1* have been identified in both AD and AR hereditary forms. However, functional differences between AD and AR mutations remain unclear. Motta et al. suggests that *LZTR1* AD mutants might work as dominant-negative forms of LZTR1 WT (17), but the molecular mechanism had not been clearly understood. In this study, we hypothesized that AD mutants lose the ability to bind to the RAS subfamily members and function as dominant-negative forms of LZTR1 WT. The AD mutations selected in this study are located in the Kelch domain of LZTR1. Structural analysis showed that amino acid residues R283 and R412 are involved in ionic interactions with RIT1, and replacing G248 with more hydronic and bulkier residues destabilizes the interaction between LZTR1 and RIT1 (32). These mutants lost the ability to interact with RIT1 and proteolyze RAS subfamily proteins (Figure 2). LZTR1 AD mutants partially recovered the LZTR1 WT–mediated inhibition of ELK1 transcriptional activity in an expression level–dependent manner (Figure 2F). This was supported by our observation that haploinsufficient mice (*Lztr1*^+/–^) did not exhibit any significant phenotypes, whereas heterozygous AD mutations caused cardiac hypertrophy and other phenotypes (Figure 1, Figure 3, and Supplemental Figures 4 and 6). Our findings suggest that LZTR1 AD mutants function as dominant-negative forms of the WT protein and that only 1 heterozygous LZTR1 AD mutant is sufficient to cause NS.

RASopathies are caused by the activation of the RAS/MAPK signaling pathway. However, it is difficult to detect the activation of the pathway in most mouse models (33–35, 52). In this manuscript, the phospho-MEK1/2 and phospho-ERK1/2 levels were slightly increased in MEFs from *Lztr1* AD mutant mice under 10% serum conditions. In mouse hearts, Western blot analysis does not show the substantial increases of phospho-MEK1/2 and phospho-ERK1/2 levels, although GSEA shows MAPK activation. The RNA-Seq results also show that the expression of *Spry4*, a negative feedback loop related gene of the MAPK signaling pathway, was upregulated in *Lztr1*^R409C/+^ mouse hearts (Supplemental Table 6). The results suggest that there might be a feedback loop in the hearts of LZTR1 AD mutant mice, and this can explain part of the reason why MAPK activation was not detected in Western blotting of heart samples. To elucidate the details of the negative feedback loop in vivo, more comprehensive data regarding LZTR1 and the RAS/MAPK signaling pathway are required. The substrate specificity of LZTR1 has not been clearly understood. It is well known that noncanonical RAS proteins, including RIT1 and MRAS, are major targets of LZTR1 (8, 11, 53). Our results from MEFs and in vitro overexpression experiments suggest that KRAS is one of the targets of LZTR1. However, KRAS expression levels were unchanged in mouse hearts (Figure 3). The results suggest the possibility of tissue specificity in the recognition of the RAS subfamily by LZTR1. Intriguingly, our previous reports indicate that LZTR1 interacted with RAF1, SHOC2, and PPP1CB and that their expression levels were not altered upon LZTR1 overexpression except for the RAS subfamily (7, 13). Based on our results and previous reports, these interacting proteins are part of the LZTR1/RAS complex and are not subjected to proteolysis by LZTR1. We have hypothesized that LZTR1 also works as a scaffold protein to facilitate the interaction between the RAS subfamily with the RAF1/SHOC2/PPP1CB complex. Further studies on tissue-specific substrates of LZTR1 and the interaction with the RAF1/SHOC2/PPP1CB complex will elucidate the negative feedback loop mechanism and help develop more efficient RAS-targeting drugs with fewer side effects for tissue-specific treatment.

The proteostasis of RAS subfamily members and other molecules regulated by LZTR1 is becoming increasingly apparent. However, the proteostatic mechanism of LZTR1 has not yet been elucidated. We show that LZTR1 was proteolyzed through the proteasome pathway and that the LZTR1 p.R412C mutation had a longer half-life than WT or other AD mutants, resulting in the accumulation of the human p.R412C/mouse p.R409C mutant in cells (HEK293 and MEFs) and in the mouse heart. Considering that LZTR1 AD mutants work in dominant-negative forms, a high expression of the LZTR1 p.R409C mutant might induce more severe phenotypes than p.G245R mutant mice. Nevertheless, *Lztr1*^R409C/+^ and *Lztr1*^G245R/+^ mice exhibited similar phenotypes in their early life stages, as evidenced by the embryos and hearts of 12-week-old mice. The transient expression of AD mutants did not result in functional differences in RAS proteostasis. On the other hand, the differences between LZTR1 AD mutant mice at later life stages remain unclear. The disruption of proteostasis induces cardiac defects and cardiovascular diseases, resulting in long-term endoplasmic reticulum stress, the unfolded protein response, and cellular senescence (54, 55). Therefore, further studies are needed to clarify the influence of LZTR1 p.R412C mutation accumulation in predicting the long-term prognosis of patients.

In summary, we identified that heterozygous AD mutations in *LZTR1* cause NS and that the mutants work as dominant-negative forms of LZTR1 WT in regulating the MAPK signaling pathway. Furthermore, we found that the MEK inhibitor trametinib was an effective drug therapy for cardiac hypertrophy in NS mice with heterozygous *LZTR1* mutation. We believe our findings open new avenues for investigating the pathogenicity of disorders with LZTR1 mutations and developing therapeutic drugs for these diseases.

## Methods

### Sex as a biological variable.

We have confirmed that female *Lztr1*^R409C/+^mice show NS-like phenotypes, including cardiac hypertrophy. To prevent the possibility that the estrous cycle might affect the body weight of female mice, we only used male mice to assess LZTR1 mutation–dependent phenotypes.

### Generation of Lztr1^R409C/+^ and Lztr1^G245R/+^ mice and animal studies.

*Lztr1*^R409C/+^ and *Lztr1*^G245R/+^ mice were generated using the CRISPR/Cas9 system with gRNA and ssDNA, as described previously (56, 57). Two sets of guide RNA and single-stranded DNA were used as single-base substitutions (Supplemental Table 1). *Lztr1*^+/–^ mice were generated as previously reported (31). These mice were backcrossed for over 8 generations with C57BL/6J mice. C57BL/6J mice were purchased from The Jackson Laboratory. The mice were weaned 4 weeks after birth and maintained under a 12-hour/12-hour light-dark cycle.

Before initiating the animal studies, the mice were randomized for the experiments. As shown in Figure 6, mice were fed 5 ppm trametinib- or 10 ppm rapamycin-containing diets for 8 weeks after weaning, and the collected tissues were used for each analysis. The amount of drug contained was equivalent to a 25 g mouse receiving 1 mg/kg/day of trametinib or 2 mg/kg/day of rapamycin, assuming a daily food intake of 5 g for adult mice.

### Western blot analyses.

Whole-cell lysates were prepared using RIPA buffer (50 mM Tris-HCl [pH 7.5] [Sigma-Aldrich, T1503], 150 mM NaCl [Wako Pure Chemical Industries, 191-01665], 1% Triton X-100 [Sigma-Aldrich, T8787], 0.5% sodium deoxycholate [Wako Pure Chemical Industries, 190-08313], and 0.1% SDS [Sigma-Aldrich, L3771]) and CelLytic M Cell Lysis Reagent (Sigma-Aldrich, C2978), for in vivo and in vitro experiments, respectively. Western blotting was performed using specific antibodies as described previously (31). The antibodies used are as follows: HA-tag (catalog 3724), phospho-ERK1/2 (catalog 9101), ERK1/2 (catalog 9102), phospho-MEK1/2 (catalog 9154), MEK1/2 (catalog 8727), phospho-AKT Ser473 (catalog 4060), phospho–AKT Thr308 (catalog 13038), AKT (catalog 9272), and GAPDH (catalog 2118) were purchased from Cell Signaling Technology; FLAG-tag (catalog F1804), RIT1 (catalog HPA053249), and β-actin (catalog A5316) were purchased from Sigma-Aldrich; LZTR1 (catalog sc-390166), NRAS (catalog sc-31), and CUL3 (catalog sc-166110) were from Santa Cruz Biotechnology Inc.; KRAS (catalog OP24) and pan-RAS (catalog 05-516) were purchased from Merck Millipore; and HRAS (catalog 18295-1-AP) and MRAS (catalog ab176570) were purchased from Proteintech and Abcam, respectively.

### Histological and IHC analyses.

Histological and IHC sections were prepared from the collected tissues as described previously (58) and stained with H&E, Masson’s trichrome (MT) stain, and TUNEL staining kit (Takara Bio, MK500) according to the manufacturer’s protocols. The deparaffinized sections were stained with WGA Alexa Fluor 594 conjugate (WGA-594; Thermo Fisher Scientific, W11262) to calculate the cross-sectional areas using ImageJ Fiji (NIH). Antigen-activated paraffin sections were stained with anti–Ki-67 antibodies (418071; Nichirei Bioscience). Wholemount immunostaining of embryonic back skin was performed as described previously (59).

### RNA-Seq analysis and DIA proteomic analysis.

Twelve-week-old mice were perfused with ice-cold phosphate-buffered saline, and their LVs were obtained for multi-omics analysis. Mouse hearts were lysed using TRIzol reagent (Thermo Fisher Scientific, 15596026), and total RNA and protein were extracted. Total RNA from perfused hearts was cleaned up with the RNeasy Mini kit (QIAGEN, 74106) and sequenced using a NEBNext Ultra II RNA Library Prep Kit for Illumina and Novaseq6000. Trim-galore was used to filter out low-quality reads and adapters from the fastq files of the RNA-Seq raw data. After quality checking, the data were aligned to the mouse GRCm39 genome using HISAT2. To analyze alterations in mRNA levels, rRNA data were excluded. The data were analyzed using the StringTie-edgeR pipeline. GSEA was performed with gene set sizes restricted to a minimum of 10 and a maximum of 500 genes using various R packages, including clusterProfiler, pathview, goProfiles, and enrichplot. The total protein was subjected to DIA proteomic analysis at the Kazusa DNA Research Institute (Kisarazu, Japan) (60, 61). Raw data were analyzed using DIA-NN and Perseus (62–64). Enrichment analyses of multiple DEPs were performed using Metascape software (65), and the relevant enrichment patterns across multiple protein lists and top enriched clusters are represented.

### Cell culture.

HEK293 cells (CRL-1573) were purchased from the American Type Culture Collection. A LZTR1-KO cell line (HEK293-KO) was generated as previously described (11). MEFs were generated from E13.5 embryos as previously described (31). Cells were cultured at 37°C in a 5% CO_2_ atmosphere in DMEM (Thermo Fisher Scientific, 11995073) supplemented with 10% FBS, Antibiotic-Antimycotic (Thermo Fisher Scientific, 15240062), and MEM Non-Essential Amino Acids Solution (Thermo Fisher Scientific, 11140050).

### Expression plasmids.

The LZTR1 expression plasmid was constructed as previously reported (11, 15). The cDNA was mutated via PCR using specific primer sets (Supplemental Table 2) and a KOD-Plus-Neo (Toyobo, KOD-401). We performed an LR recombination reaction to generate expression plasmids and transferred the gene of interest into pcDNA3.2 (V5-tag) using Gateway technology.

### Protein interaction assays.

Recombinant proteins were generated using a TnT T7 Quick Coupled Transcription/Translation System (Promega, L1170). In Figure 2D, in vitro*–*translated FLAG-tagged LZTR1 proteins were incubated with HEK239-KO whole-cell lysates and FLAG-M2 magnetic beads (Sigma-Aldrich, M8823), and the molecular interaction between LZTR1 mutants and RAS subfamily was examined via Western blotting. In Figure 2E, in vitro–translated proteins were mixed at the indicated protein ratios and incubated with FLAG-M2 magnetic beads for 6 hours. The immunoprecipitants were subjected to Western blot analysis.

### Statistics.

Statistical analyses were performed using the R software package. In vitro and in vivo data are presented as the mean ± SD and mean ± SEM*,* respectively. Significant differences between the control and treatment groups were assessed using the Wilcoxon–Mann–Whitney test, Dunnett’s test, or log-rank test, where applicable. A *P* value less than 0.05 was considered significant.

### Study approval.

All animal experiments were approved by the Animal Care and Use Committee of Tohoku University (2020MdA-097-06, 2020MdA-095-04, 2020MdA-100-05) and performed in accordance with the guidelines for animal experimentation at Tohoku University.

### Data availability.

RNA-Seq data were deposited in the Gene Expression Omnibus (GEO) database (accession no. GSE254067; https://www.ncbi.nlm.nih.gov/geo/query/acc.cgi?acc=GSE254067). DIA proteomic analysis data were deposited in ProteomeXchange Consortium via the jPOST partner repository under the accession number (PXD048793, JPST002472; https://repository.jpostdb.org/entry/JPST002472; https://proteomecentral.proteomexchange.org/cgi/GetDataset?ID=PXD048793). The raw data values for the data presented in this manuscript are accessible in the Supporting Data Values file. All other data and materials analyzed in this study are available from the corresponding author upon reasonable request.

## Author contributions

TA and YA designed the experiments. TA and KM performed the in vivo experiments. TA performed the in vitro experiments and analyzed RNA-Seq and proteome data. TN supported the data analysis of RNA-Seq. MT and ST created the genetically modified mice. TA and YA obtained funding for the study. TA wrote the draft. TA, TN, and YA revised the manuscript.

## Supplementary Material

Supplemental data

Unedited blot and gel images

Supplemental table 3

Supplemental table 4

Supplemental table 5

Supplemental table 6

Supporting data values

## Version 1 10/01/2024

In-Press Preview

## Version 2 11/22/2024

Electronic publication
