# Supplementary material for: Dysregulation of RAS proteostasis by autosomal-dominant LZTR1 mutation induces Noonan syndrome–like phenotypes in mice
Source: JCI Insight. 2024 Nov 22;9(22):e182382. doi: 10.1172/jci.insight.182382 (PMC11601938; doi:10.1172/jci.insight.182382)
Supplement: Supplemental data [file jciinsight-9-182382-s089.pdf]

Supplementary Materials

Supplementary Figures

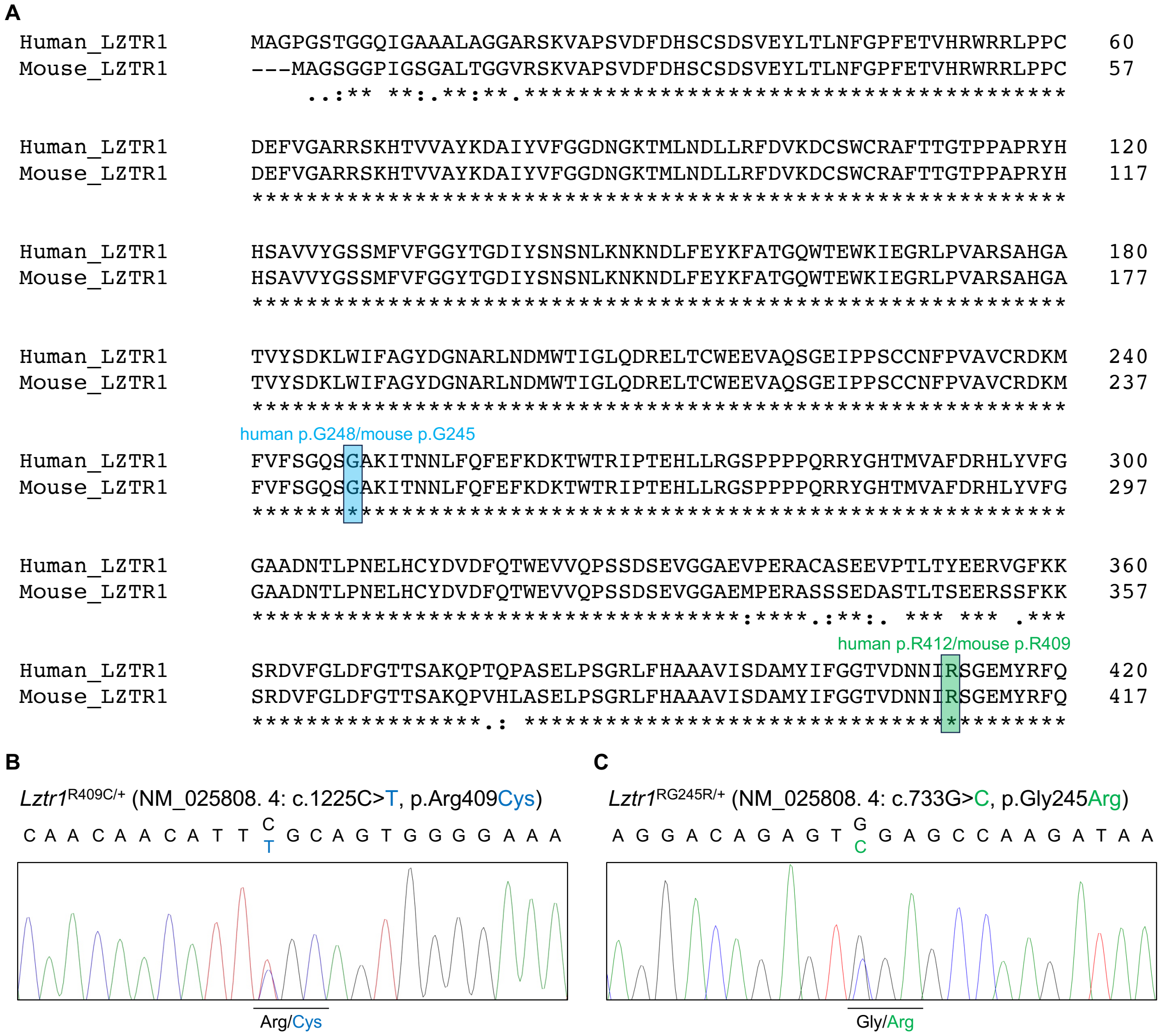

Supplementary Figure 1. Gene-mutated sites in *Lztr1* mutant mice.

Graphical representation of the homology of the LZTR1 protein sequence between humans and mice. Blue and green markers show human p.G248/mouse p.G245 and human p.R412/mouse p.R409 sites, respectively.

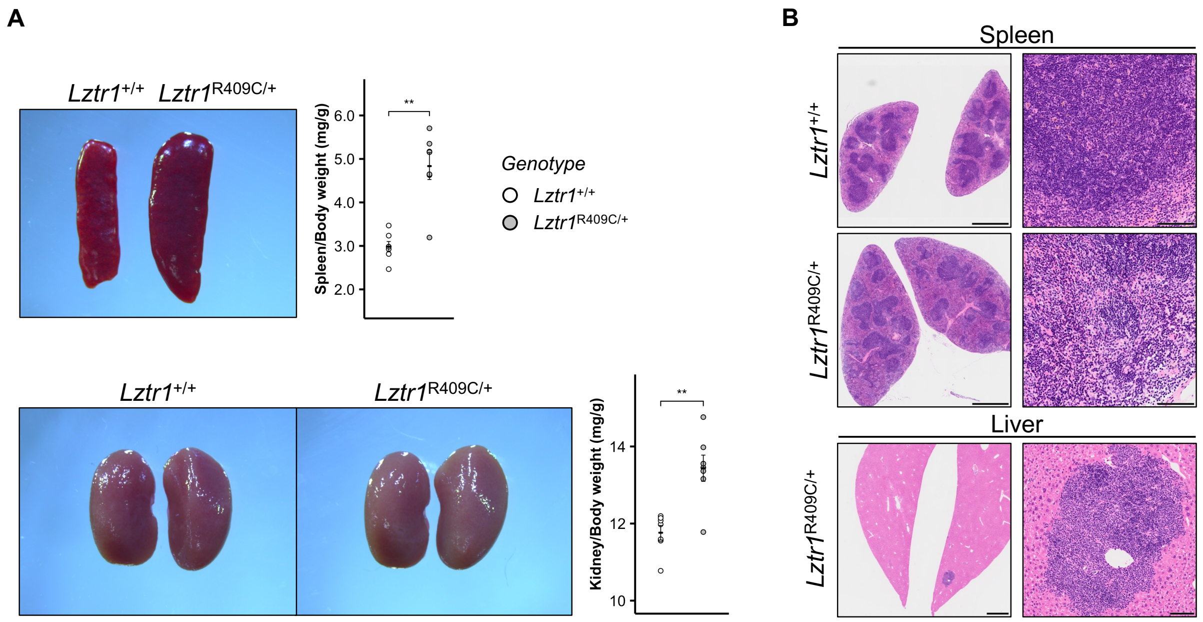

Supplementary Figure 2. *Lztr1*$^{R409C/+}$ mice showed splenomegaly and renal hypertrophy.

Samples were collected from *Lztr1*$^{R409C/+}$ and *Lztr1*$^{+/+}$ male mice at 12 weeks of age.

(**A**)Spleen or kidney-to-body weight ratio was calculated. Values are presented as mean ± SEM (n = 6). $^{**}p \leq .01$ (Wilcoxon-Mann-Whitney test). (**B**) HE-stained sections of spleen and liver. Scale bars indicate 1 mm or 100 μm.

A

♂ *Lztr1*$^{R409C/+}$ × ♀ *Lztr1*$^{+/+}$	Weaning
Lztr1$^{+/+}$	338 (62.5%)
Lztr1$^{R409C/+}$	203 (37.5%)

B

♂ *Lztr1*$^{R409C/+}$ × ♀ *Lztr1*$^{R409C/+}$	E14.5	E16.5	E18.5	Weaning (P28)
Lztr1$^{+/+}$	13	7	8	2
Lztr1$^{R409C/+}$	6	16	12	9
Lztr1$^{R409C/R409C}$	8	4	0	0
Total	27	27	20	11

C

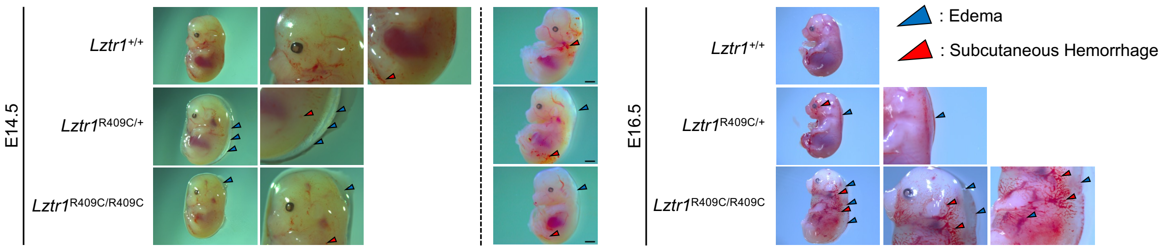

E14.5	Edema	Subcutaneous Hemorrhage
Lztr1$^{+/+}$	0/13 (0%)	3/13 (23%)
Lztr1$^{R409C/+}$	5/6 (83%)	5/6(83%)
Lztr1$^{R409C/R409C}$	8/8 (100%)	6/8 (75%)

E16.5	Edema	Subcutaneous Hemorrhage
Lztr1$^{+/+}$	0/7 (0%)	1/7 (14%)
Lztr1$^{R409C/+}$	8/16 (50%)	8/16 (50%)
Lztr1$^{R409C/R409C}$	4/4 (100%)	4/4 (100%)

D

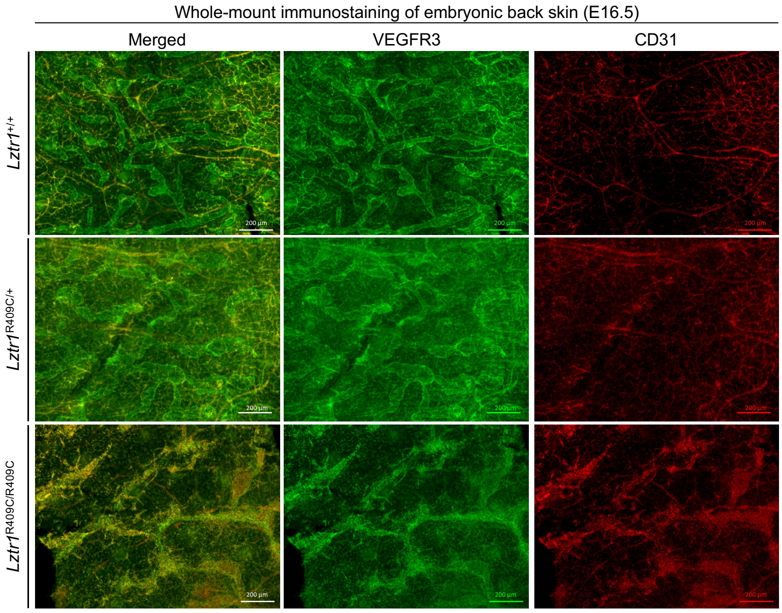

E

♂ *Lztr1*$^{G245R/+}$ × ♀ *Lztr1*$^{+/+}$	Weaning
Lztr1$^{+/+}$	125 (63.5%)
Lztr1$^{G245R/+}$	72 (36.5%)
Total	197

F

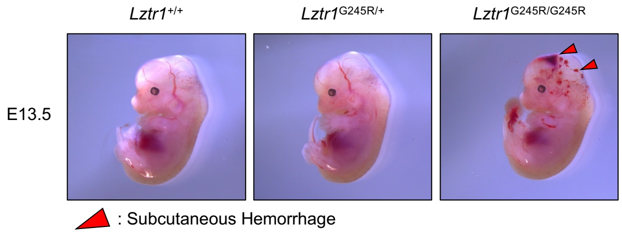

Supplementary Figure 3. Embryonic analysis of *the Lztr1*$^{R409C/+}$ mice.

(**A**) Genotypes of pups obtained from intercrosses between male *Lztr1*$^{R409C/+}$ and female *Lztr1*$^{+/+}$ mice. We used the mice for analyzing their postnatal phenotypes. (**B**) Genotypes of pups and embryos from intercrosses between male *Lztr1*$^{R409C/+}$ and female *Lztr1*$^{R409C/+}$ mice. (**C**) Representative images of embryos at E14.5 and E16.5. Blue and red arrowheads indicate edema and subcutaneous hemorrhage, respectively. (**D**) Whole-mount immunostaining of embryonic back skins was performed using E16.5 embryos from *Lztr1*$^{+/+}$, *Lztr1*$^{R409C/+}$, *Lztr1*$^{R409C/R409C}$. The back skins were stained with anti-VEGFR3 antibody (green) and anti-CD31 (red) antibody. Scale bar, 200 μm. (**E**) Genotypes of pups from the intercrosses between male *Lztr1*$^{G245R/+}$ and female *Lztr1*$^{+/+}$ mice. (**F**) Representative images of the embryos at E13.5. Blue and red arrowheads indicate edema and subcutaneous hemorrhage, respectively.

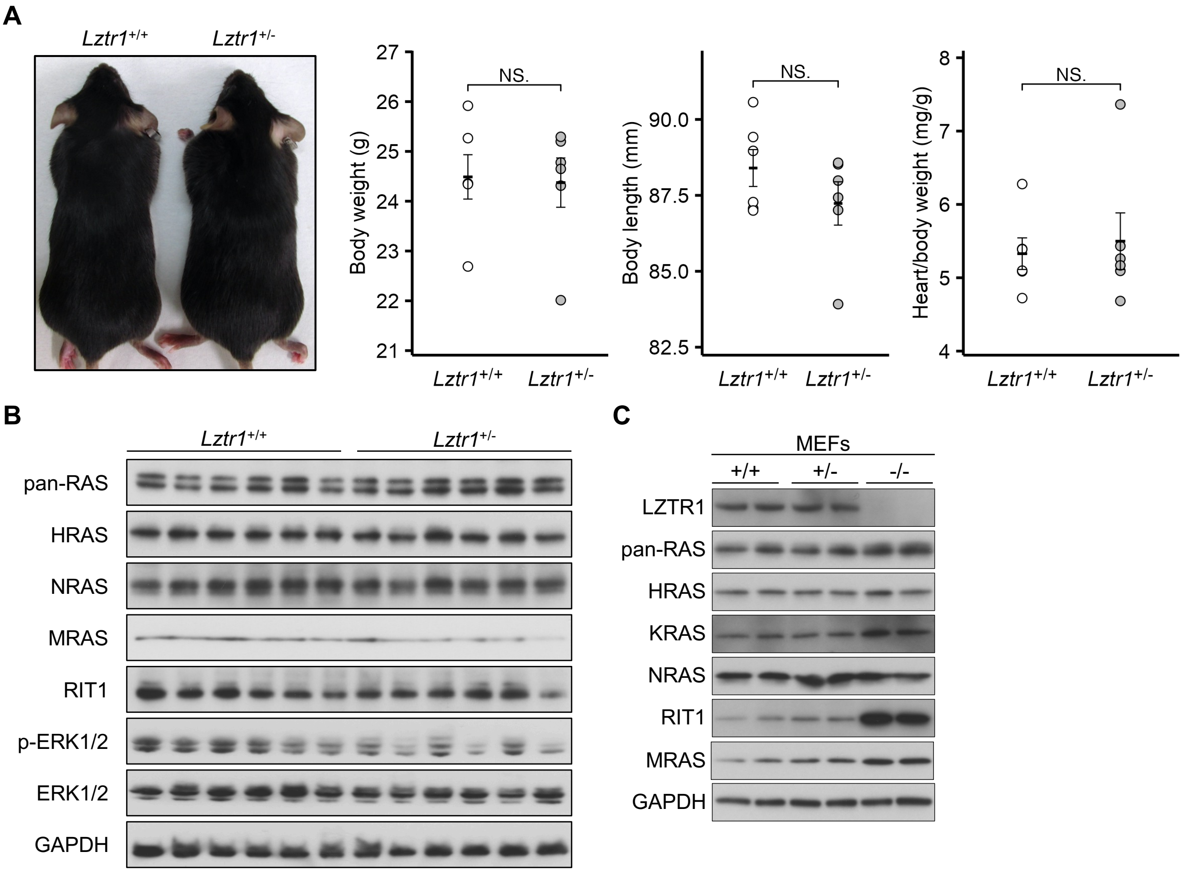

Supplementary Figure 4. *Lztr1*$^{+/-}$ mice exhibited no NS-like phenotypes.

(**A**) Representative images of *Lztr1*$^{+/-}$ and *Lztr1*$^{+/+}$ male mice at 12 weeks of age. Body weight, body length, and heart-to-body weight ratio were calculated. Values are presented as the mean ± SEM (n = 6). NS: not significant (Wilcoxon-Mann-Whitney test). (**B, C**) Immunoblot analysis of LVs or MEFs was performed using the indicated antibodies.

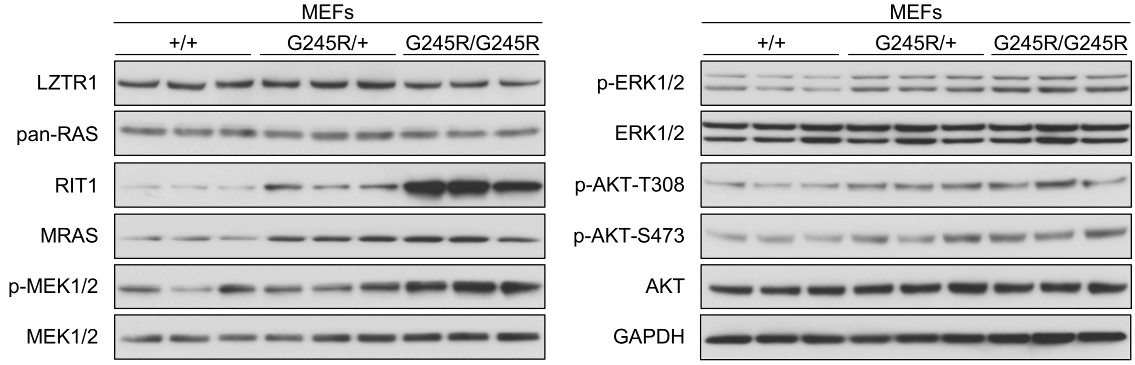

Supplementary Figure 5. The analysis of RAS expression levels in MEFs from *Lztr1*$^{+/+}$, *Lztr1*$^{G245R/+}$, *and Lztr1*$^{G245R/G245R}$ embryos.

MEFs from *Lztr1*$^{+/+}$*, Lztr1*$^{G245R/+}$*, and Lztr1*$^{G245R/G245R}$ embryos at E13.5 were analyzed using the indicated antibodies.

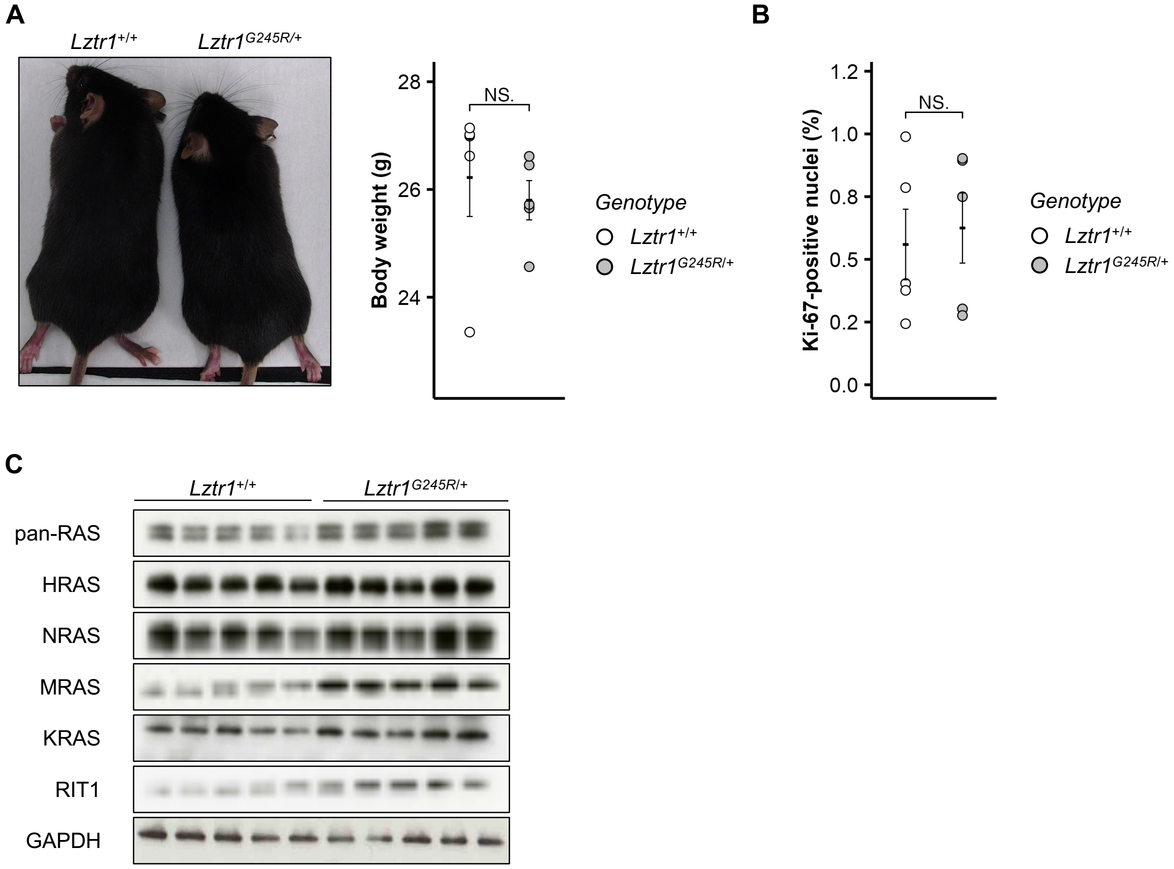

Supplementary Figure 6. *Lztr1*$^{\mathrm{G245R/+}}$ mice exhibited NS patient-like phenotypes.

Samples were collected from *Lztr1*$^{\mathrm{G245R/+}}$ and *Lztr1*$^{+/+}$ male mice at 12 weeks of age. (**A**) Representative images of mice. The body weight was calculated at 12 weeks of age. (**B**) Paraffin-embedded sections were stained with anti Ki-67 antibody. The Ki-67 positive nuclei was calculated using the ImageJ Fiji. Values are presented as the mean ± SEM (n = 5). *$p \leq .05$, **$p \leq .01$, NS: not significant (Wilcoxon-Mann-Whitney test). (**C**) Immunoblot analysis was performed using the indicated antibodies.

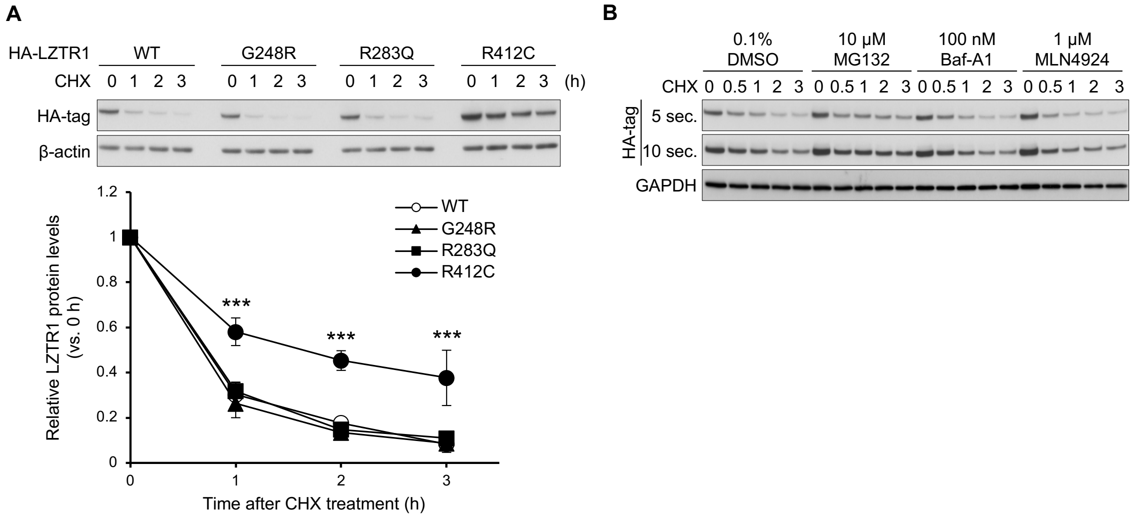

Supplementary Figure 7. Cycloheximide chase assay to estimate the protein stability of LZTR1.

(**A**) HEK293 cells were transfected with the indicated plasmids and treated with 50 μg/ml cycloheximide for 1-3 hours. LZTR1 protein levels were evaluated by immunoblotting with anti-HA-tag and anti-GAPDH antibodies. Values are presented as mean ± SEM (n = 3). ***$p \leq .001$ (vs. WT group, Wilcoxon-Mann-Whitney test). (**B**) HEK293 cells were transfected with HA-LZTR1-pcDNA and then treated with 50 μg/ml cycloheximide with 0.1% DMSO, 10 μM MG132, 100 nM bafilomycin A1 (Baf-A1), or 1 μM MLN4924. LZTR1 protein levels were evaluated by immunoblotting, as shown in Fig. S8A.

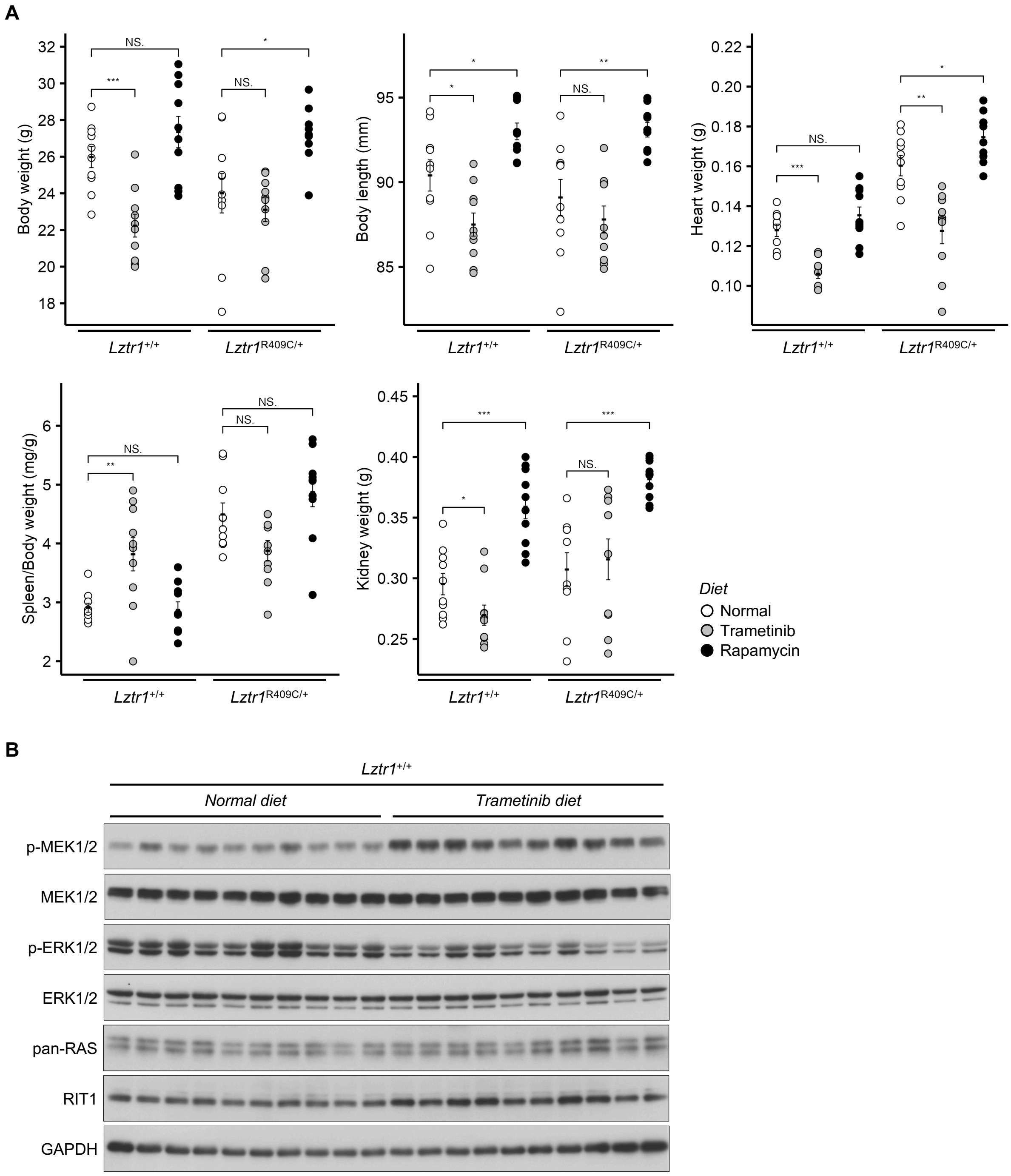

Supplementary Figure 8. Effect of treatment with trametinib and rapamycin in *Lztr1*$^{\mathrm{R409C/+}}$ and *Lztr1*$^{+/+}$ mice.

Mice were fed 5 ppm trametinib- or 10 ppm rapamycin-containing diets for 8 weeks after weaning, and the collected tissues were used for each analysis. (**A**) Measurement of body weight, body length, and weight of each tissue. Values are presented as mean ± SEM (n =

10). *$p \leq .05$, **$p \leq .01$, ***$p \leq .001$ NS: not significant (Wilcoxon-Mann-Whitney test). (**B**) Immunoblot analysis was performed with the indicated antibodies using LVs from *Lztr1*$^{+/+}$ mice.

Supplementary Tables

Supplementary Table 1. The gRNAs and ssDNAs were used for mouse generation.

Name	Sequence (5′ to 3′)
gRNA for *Lztr1*$^{R409C/+}$	GTAGACAACAACATTCGCAG<u>TGG</u>
ssDNA for *Lztr1*$^{R409C/+}$	ACTAGCTACTCACCTGGAACCTGTACATTTCCCCACTGCAAATGTTGTTGTCTACAGTGCCCC CAAAGATGTACATGGC
gRNA for *Lztr1*$^{G245R/+}$	TTCGTGTTCTCAGGACAGAG<u>TGG</u>
ssDNA for *Lztr1*$^{G245R/+}$	TGAATTCAAACTGGAAGAGGTTGTTAGTTATCTTGGCTCGACTCTGTCCTGAGAACACGAAC ATCTTATCCCGGCACAC

The protospacer-adjacent motif is underlined in the gRNA sequences.

Supplementary Table 2. Primers used for construct preparation.

Name	Sequence (5′ to 3′)	Reverse primer (5′ to 3′)
LZTR1-R412C_mutagenesis	TGCAGCGGGGAGATGTACAGGTTCC	GATGTTGTTGTCCACCGTGCCCCCG
LZTR1-R412H_mutagenesis	ACAGCGGGGAGATGTACAGGTTCC	GGATGTTGTTGTCCACCGTGCCCCCG
