# Supplementary material for: Dysregulation of RAS proteostasis by autosomal-dominant LZTR1 mutation induces Noonan syndrome–like phenotypes in mice
Source: JCI Insight. 2024 Nov 22;9(22):e182382. doi: 10.1172/jci.insight.182382 (PMC11601938; doi:10.1172/jci.insight.182382)

Full unedited blot for Fig. 2A

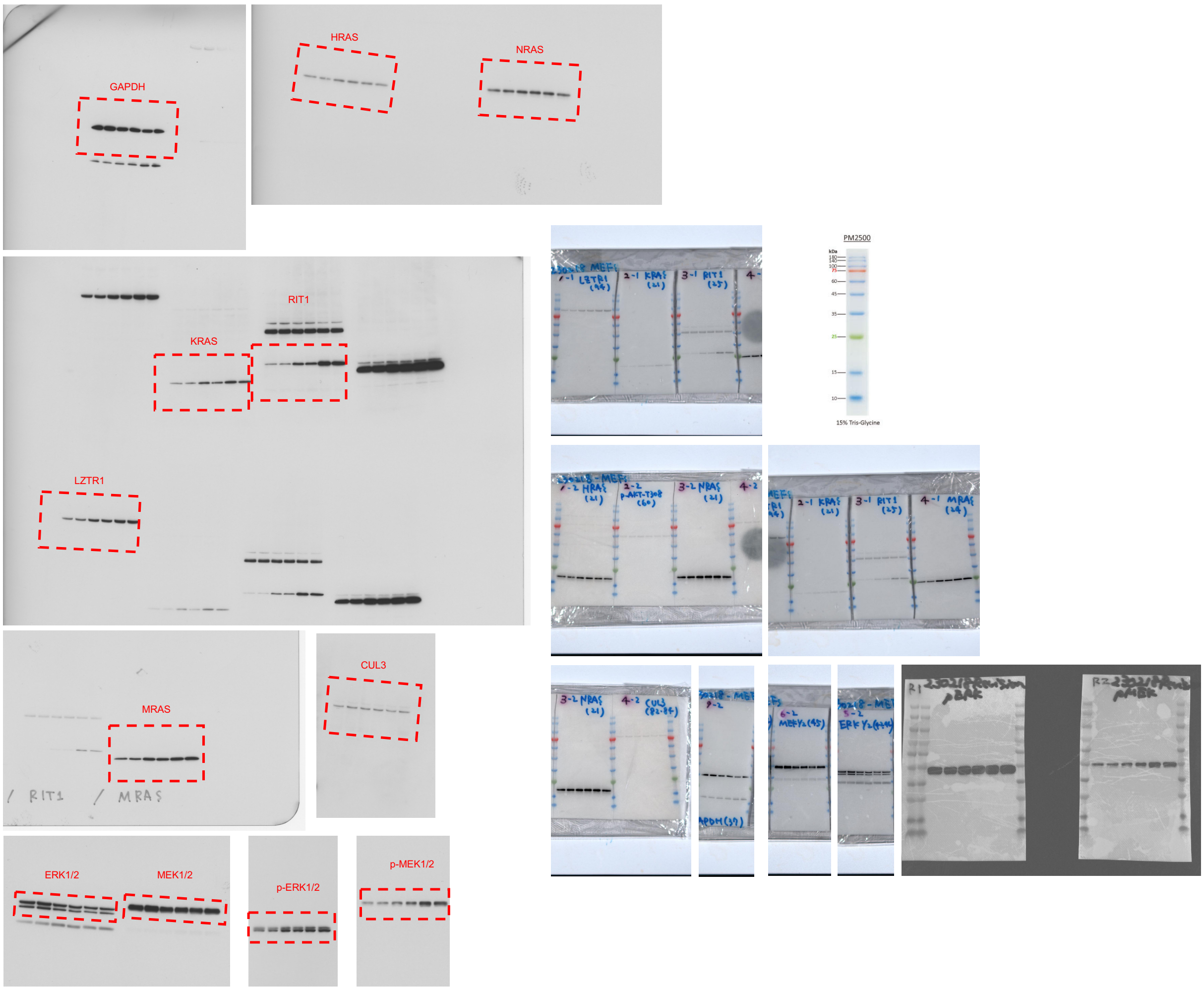

Full unedited blot for Fig. 2B

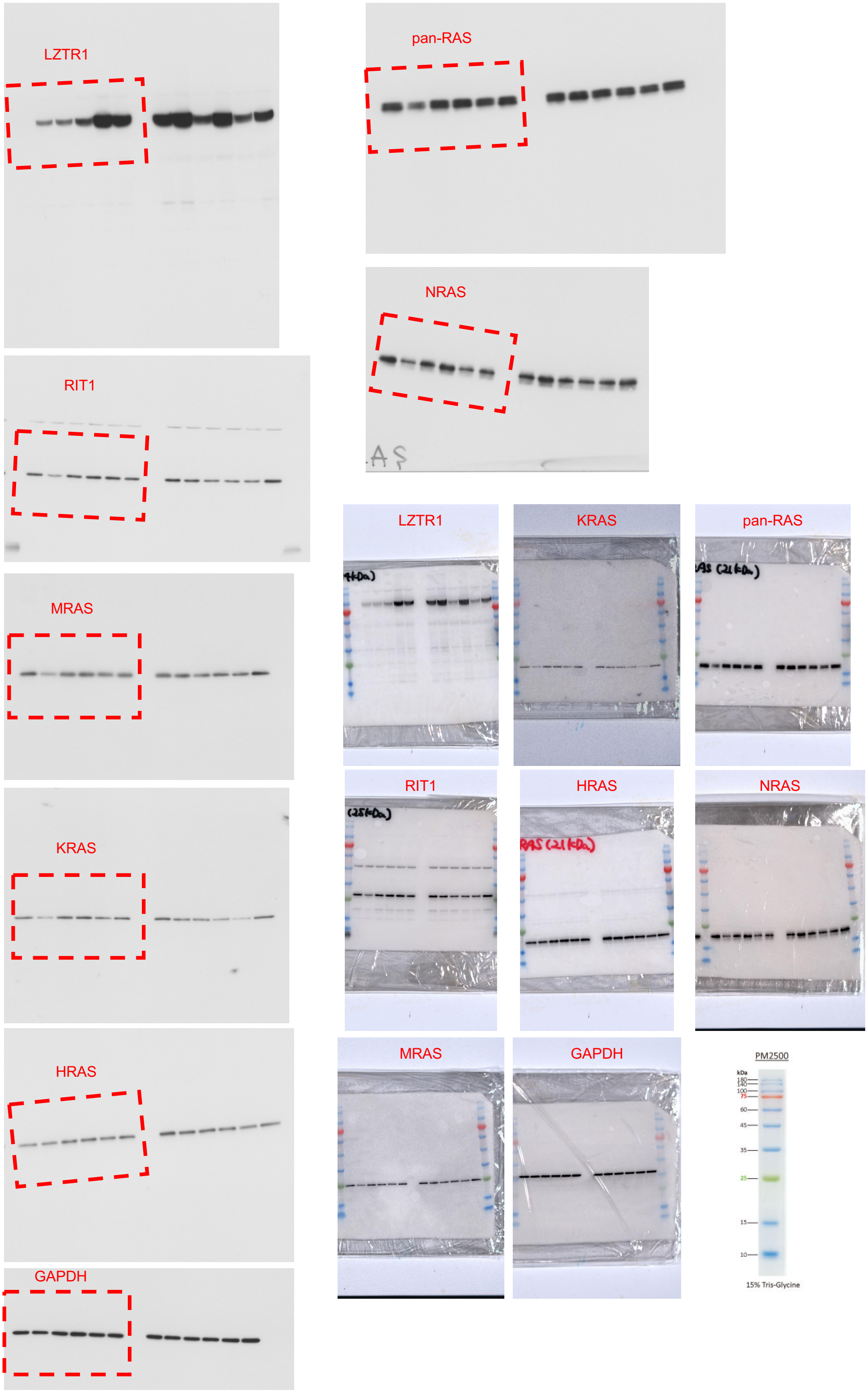

Full unedited blot for Fig. 2D

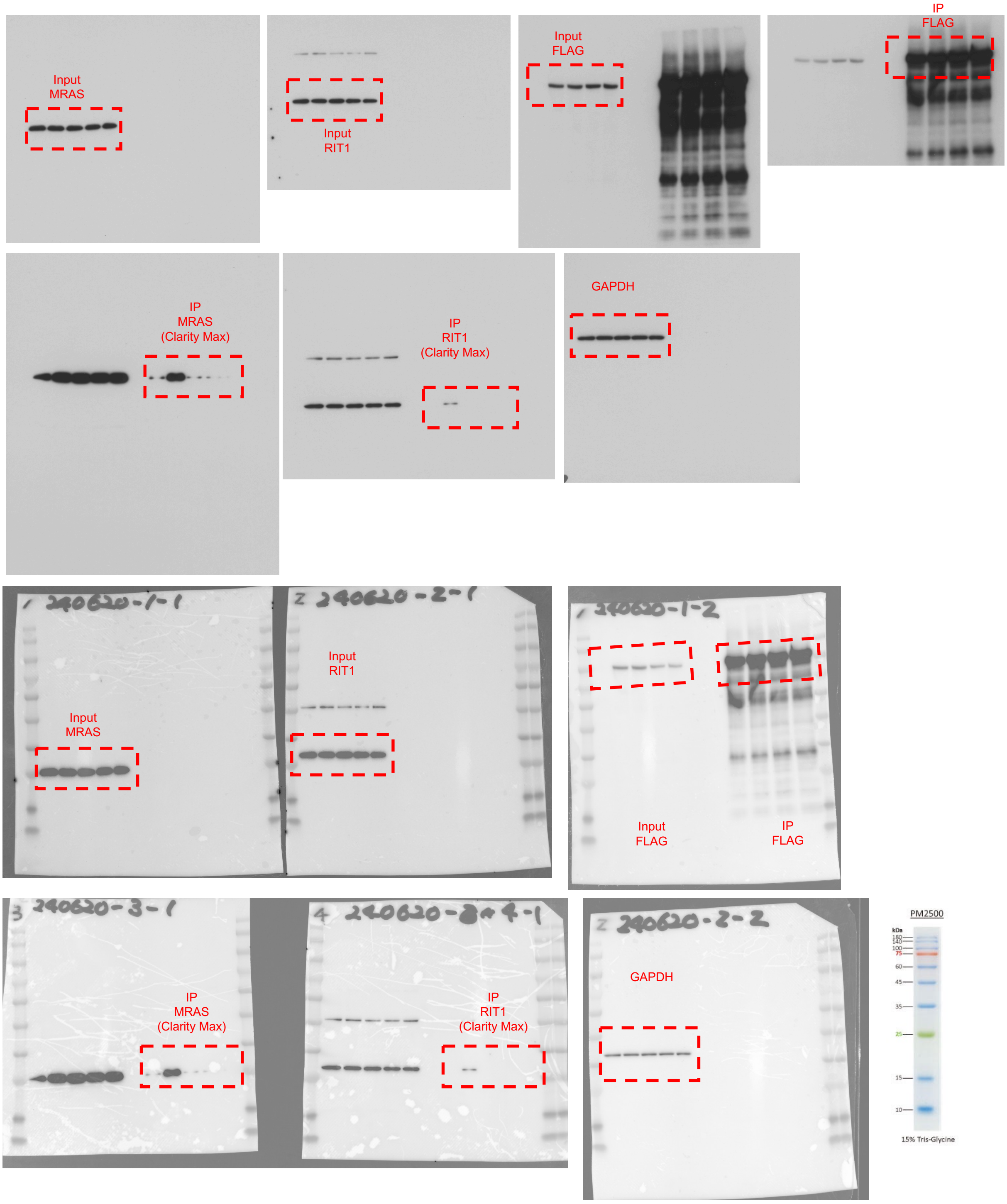

Full unedited blot for Fig. 2E

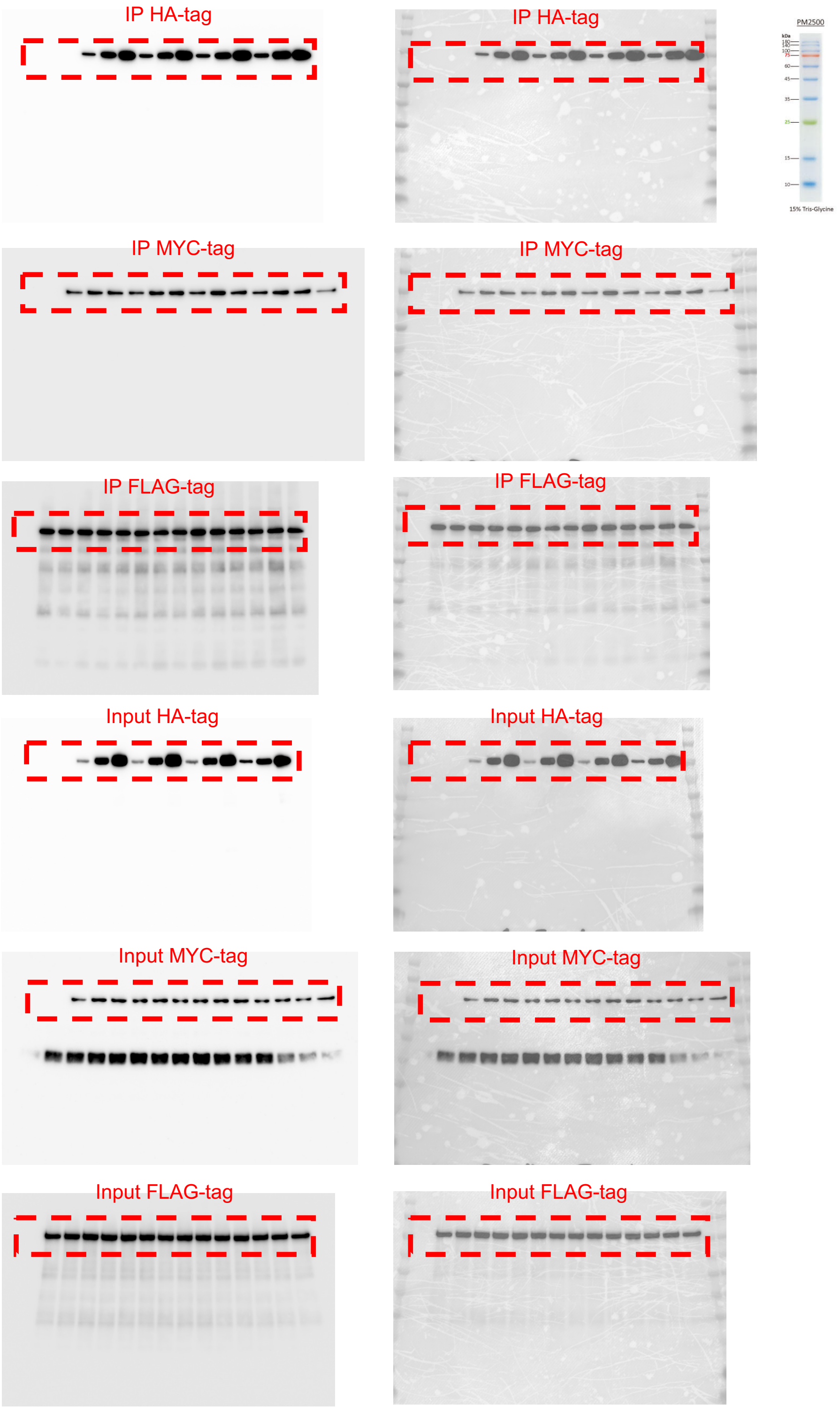

Full unedited blot for Fig. 3B

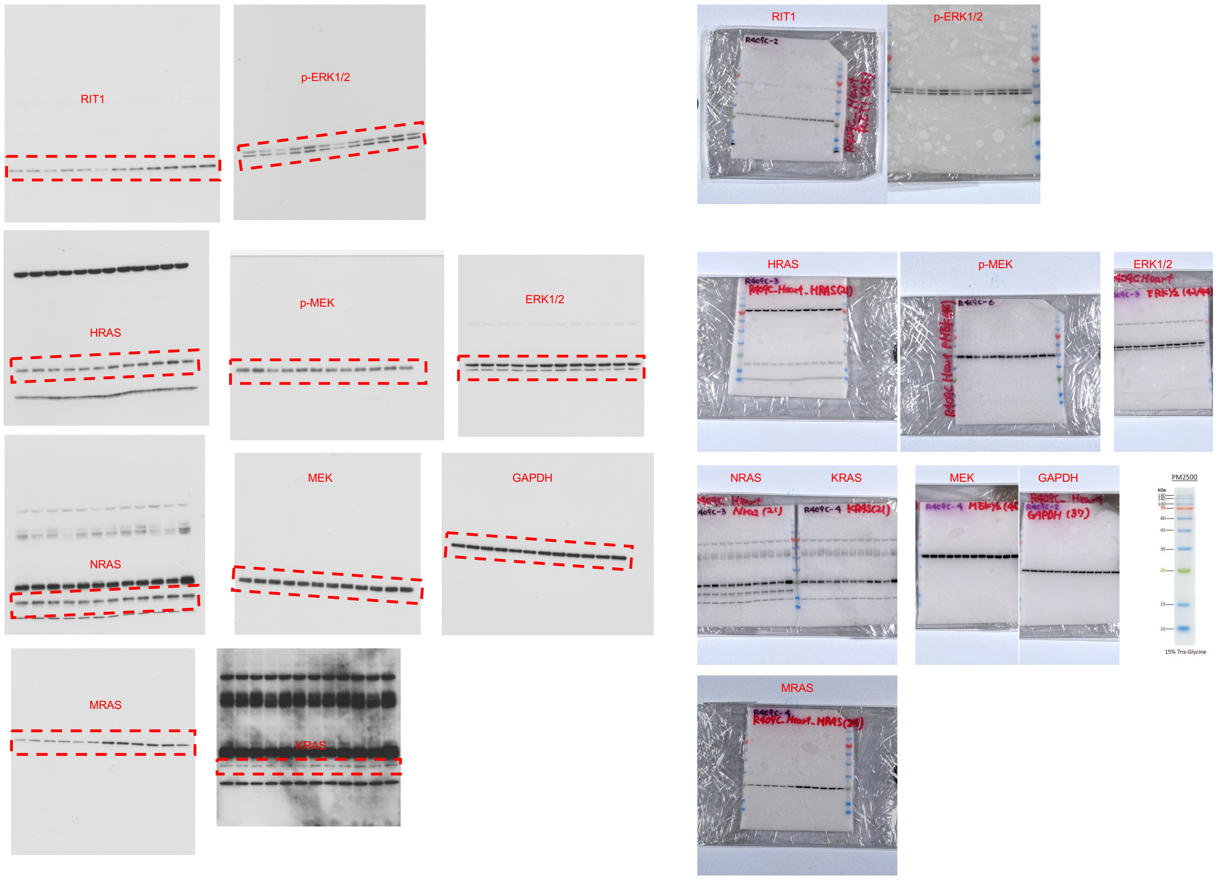

Full unedited blot for Fig. 6C

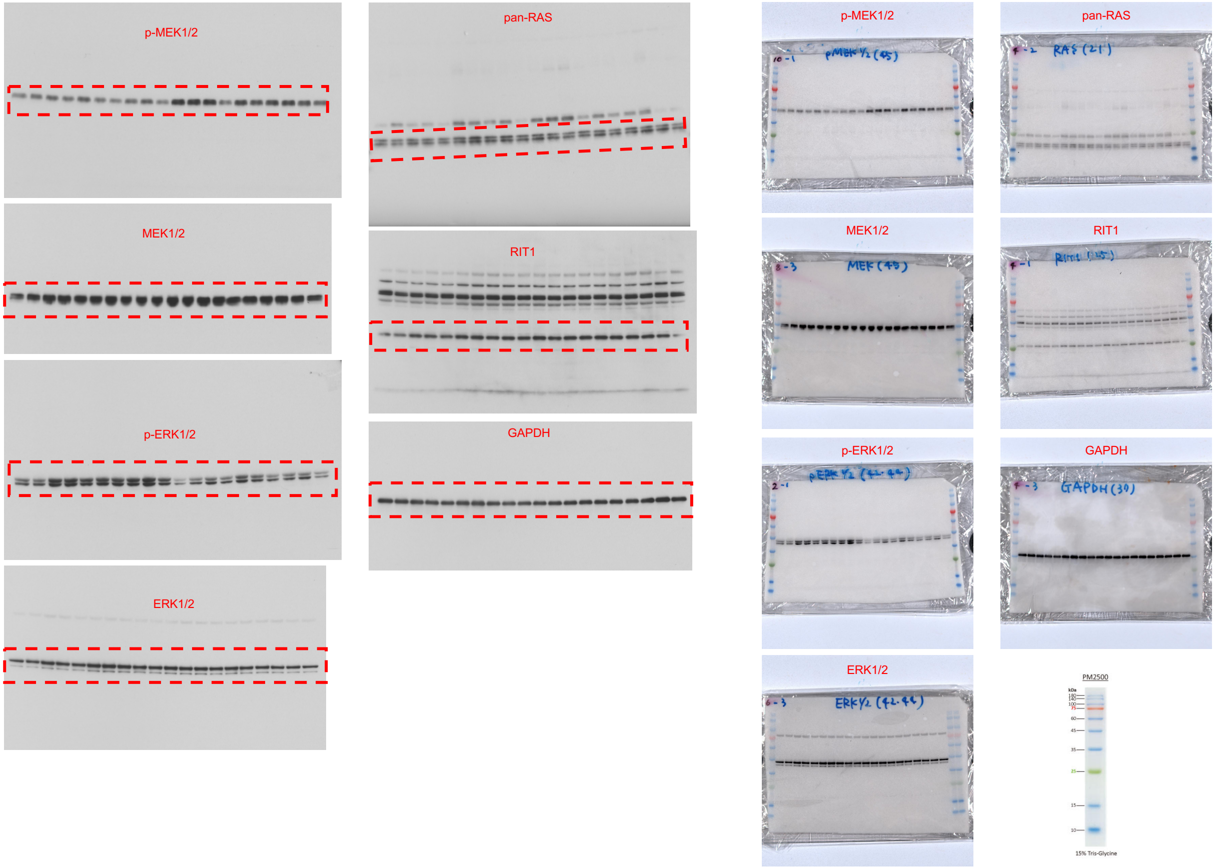

Supplement: Unedited blot and gel images [file jciinsight-9-182382-s090.pdf]
